# Analysis of the lncRNA–miRNA–mRNA Network Reveals a Potential Regulatory Mechanism of EGFR-TKI Resistance in NSCLC

**DOI:** 10.3389/fgene.2022.851391

**Published:** 2022-04-29

**Authors:** Dandan Ding, Jufeng Zhang, Zhiming Luo, Huazhen Wu, Zexiao Lin, Weicheng Liang, Xingyang Xue

**Affiliations:** ^1^ Department of Thoracic Surgery, Affiliated Cancer Hospital & Institute of Guangzhou Medical University, Guangzhou, China; ^2^ Qingyuan People’s Hospital, The Sixth Affiliated Hospital of Guangzhou Medical University, Qingyuan, China; ^3^ Department of Medical Oncology, The Third Affiliated Hospital of Sun Yat-sen University, Guangzhou, China; ^4^ Biotherapy Center, The Third Affiliated Hospital of Sun Yat-sen University, Guangzhou, China

**Keywords:** EGFR-TKIs, drug resistance, long non-coding RNA, LINC01128, lung cancer-diagnosis

## Abstract

Epidermal growth factor receptor tyrosine kinase inhibitors (EGFR-TKIs) are widely used for patients with EGFR-mutated lung cancer. Despite its initial therapeutic efficacy, most patients eventually develop drug resistance, which leads to a poor prognosis in lung cancer patients. Previous investigations have proved that non-coding RNAs including long non-coding RNAs (lncRNAs), circular RNAs (circRNAs), and microRNAs (miRNAs) contribute to drug resistance by various biological functions, whereas how they regulate EGFR-TKI resistance remains unclear. In this study, we examined gene expression using the microarray technology on gefitinib-resistant NSCLC cells to obtain differentially expressed (DE) lncRNAs and mRNAs. A total of 45 DE-lncRNAs associated with overall survival and 1799 target DE-mRNAs were employed to construct a core lncRNA–miRNA–mRNA network to illustrate underlying molecular mechanisms of how EGFR-TKI resistance occurs in NSCLC. We found that target DE-mRNAs were mainly enriched in pathways involved in EGFR-TKI resistance, especially the target DE-mRNAs regulated by LINC01128 were significantly enriched in the PI3K/Akt signaling pathway, where the synergy of these target DE-mRNAs may play a key role in EGFR-TKI resistance. In addition, downregulated LINC01128, acting as a specific miRNA sponge, decreases PTEN *via* sponging miR-25-3p. Furthermore, signaling reactions caused by the downregulation of PTEN would activate the PI3K/Akt signaling pathway, which may lead to EGFR-TKI resistance. In addition, a survival analysis indicated the low expression of LINC01128, and PTEN is closely related to poor prognosis in lung adenocarcinoma (LUAD). Therefore, the LINC01128/miR-25-3p/PTEN axis may promote EGFR-TKI resistance *via* the PI3K/Akt signaling pathway, which provides new insights into the underlying molecular mechanisms of drug resistance to EGFR-TKIs in NSCLC. In addition, our study sheds light on developing novel therapeutic approaches to overcome EGFR-TKI resistance in NSCLC.

## Introduction

Lung cancer is the second most common malignant tumor worldwide and the leading cause of cancer-related deaths (Sung et al*.*, 2021). Non–small cell lung cancer (NSCLC) is the most common pathological type of lung cancer, accounting for 80–85% of lung cancer cases (Ettinger et al*.*, 2021). Although targeted therapies have significantly improved the prognosis of NSCLC patients harboring activating epidermal growth factor receptor (EGFR) mutations (Rosell et al*.*, 2017), NSCLC patients inevitably develop disease progression after a median of 10.2–18.9 months on EGFR-TKI treatment (Soria et al., 2018). Many mechanisms of resistance to EGFR-TKIs, such as the T790M mutation, ERBB2 amplification, MET alterations, C797X, SCLC transformation, and oncogene fusions, have been reported (Oxnard et al*.*, 2018; Piper-Vallillo et al., 2020). However, the mechanisms of resistance to *EGFR*-*TKIs* have not been fully elucidated yet. Therefore, further research studies exploring the mechanisms of EGFR-TKI resistance are still indispensable.

The competing endogenous RNA (ceRNA) mechanism indicated that lncRNAs could competitively adsorb microRNAs by sponging microRNA response elements (MRE), thereby inhibiting the function of miRNAs and regulating the expression of protein-coding genes indirectly (Salmena et al*.*, 2011). In recent years, the ceRNA regulatory network has attracted more and more attention, and previous investigations have revealed that ceRNA networks played a significant role in tumor development, including cell proliferation, metastasis, apoptosis, and chemoresistance (Yang et al., 2018; Wang et al*.*, 2019; Zhang L. et al., 2020; Zhang X.-Z. et al., 2020). For example, the long non-coding RNA LCAT1 upregulated RAC1 and PAK1 *via* competitively binding with miR-4715-5p, resulting in lung cancer cell proliferation and metastasis (Yang et al*.*, 2019). In addition, Linc00173 promoted lung cancer chemoresistance, proliferation, migration, and invasion by acting as a ceRNA for miR-218, resulting in upregulation of Etk (Zeng et al*.*, 2020). Our previous studies demonstrated that lncRNA HOTAIR could promote ER activation and drive tamoxifen resistance (Xue et al*.*, 2016). Moreover, the lncRNA HOXA11-AS could promote cisplatin resistance in lung adenocarcinoma *via* acting as a ceRNA against miR-454-3p (Zhao et al., 2018). Recently, Chen et al. demonstrated that the lncRNA H19 could act *via* upregulating PKM2 and enhancing Akt phosphorylation to modulate erlotinib resistance in EGFR-mutant lung cancer (Chen C. et al., 2020). However, there are limited research studies focused on the mechanisms of ceRNA in drug resistance to EGFR-TKIs in NSCLC.

In this study, to investigate the underlying ceRNA mechanisms of EGFR-TKI resistance in NSCLC, a differential expression analysis was performed for the expression profile of gefitinib-resistant NSCLC cells based on the microarray technology. Then we carried out a KEGG enrichment analysis for DE-mRNAs and constructed the ceRNA regulatory network of DE-lncRNAs and DE-mRNAs using starBase 2.0. Our results showed that downregulated LINC01128 acts as a miRNA sponge to reduce the PTEN expression by sponging miR-25-3p. Furthermore, the signal response caused by the reduction of the PTEN expression would activate the PI3K/Akt pathway, which may result in the development of drug resistance to EGFR-TKIs. Our findings will provide a novel perspective for the underlying molecular mechanisms of EGFR-TKI resistance in NSCLC. Moreover, this research will help to develop new therapeutic targets for EGFR-TKI resistance in NSCLC.

## Materials and Methods

### Cell Lines and Culture Conditions

PC-9 cells were obtained from Affiliated Cancer Hospital & Institute of Guangzhou Medical University. The gefitinib-resistant PC-9 cell line was established by continually exposing to a stepwise escalation of the concentration of gefitinib (Selleckchem, United States) from 0.1 to 15 μM over 8 months. Gefitinib-sensitive and gefitinib-resistant PC-9 cell lines were maintained in RPMI 1640 (Gibco, United States) supplemented with 10% fetal bovine serum (Gibco, United States) and 1% penicillin and streptomycin (Thermo Fisher Scientific) in a humidified condition with 5% CO_2_ at 37°C.

### CCK-8 Assay and Resistance Index Measure

The gefitinib-resistant PC-9 cell line and relevant parental cell line were respectively seeded in 96-well plates (3 × 10^3^ cells per well) and treated with various concentrations of gefitinib for 96 h. Each concentration was set in three replicate wells.

### RNA Extraction and Quality Control

Total RNA was isolated from cells by using the Trizol reagent, according to the manufacturer’s protocol. RNA quantity and quality were measured by using a NanoDrop ND-1000 apparatus. RNA integrity was assessed by standard denaturing agarose gel electrophoresis.

### LncRNA/mRNA Microarray

Sample labeling and array hybridization were performed according to the Agilent One-Color Microarray-Based Gene Expression Analysis protocol (Agilent Technology). In brief, mRNA was purified from total RNA after removal of rRNA (mRNA-ONLY™ Eukaryotic mRNA Isolation Kit, Epicentre). Then each sample was amplified and transcribed into fluorescent complementary RNA (cRNA) along the entire length of the transcripts without 3' bias utilizing a random priming method (Arraystar Flash RNA Labeling Kit, Arraystar). The labeled cRNAs were purified by using an RNeasy Mini Kit (Qiagen). The concentration and specific activity of the labeled cRNAs (pmol Cy3/μg cRNA) were measured by using a NanoDrop ND-1000 apparatus. In addition, 1 μg of each labeled cRNA was fragmented by adding 5 μl 10X blocking agent and 1 μl of 25X fragmentation buffer and then heating the mixture at 60°C for 30 min; then, 25 μl 2X GE hybridization buffer was added to dilute the labeled cRNA. Furthermore, 50 μl of the hybridization solution was dispensed into the gasket slide and assembled on the lncRNA expression microarray slide. The slides were incubated for 17 h at 65°C in an Agilent hybridization oven. The hybridized arrays were washed, fixed, and scanned using the Agilent DNA Microarray Scanner (part number G2505C).

### Data Processing

Agilent Feature Extraction software (version 11.0.1.1) was used to analyze acquired array images. Quantile normalization and subsequent data processing were performed using the GeneSpring GX v12.1 software package (Agilent Technologies). To identify significant probes, the Benjamini–Hochberg false discovery rate was used for the multiple testing correction p-value. Probes with a p-value < 0.05 and a fold change ≥ 2 were considered significant.

### Quantitative Real-Time Polymerase Chain Reaction

The total RNA was isolated from PC-9 and PC-9/GR using the TRIzol reagent (Invitrogen, CA), following the manufacturer’s protocol, respectively. Total RNA of 1 μg was used for reverse transcription. The designed primers were used for quantitative real-time PCR in the Bio-Rad PCR instrument; glyceraldehyde-3-phosphate dehydrogenase (GAPDH) was used as an internal control to normalize the relative gene expression by the 2^−ΔΔCt^ method. Each sample was analyzed in triplicates. The primers used in this study are as follows: LINC01128 sense: 5′-CAG​AGG​AGC​TAC​GAA​GGG​AG-3′; LINC01128 antisense: 5′-CAG​AGG​AGC​TAC​GAA​GGG​AG-3′; PTEN sense: 5′-ATG​ACA​GCC​ATC​ATC​AAA​GAG-3′; and PTEN antisense: 5′-AGG​ATA​TTG​TGC​AAC​TCT​GCA-3′.

### Differential Expression Analysis of lncRNAs and mRNAs

Differentially expressed lncRNAs and mRNAs between the two samples were identified by fold-change filtering. We detected total lncRNAs and mRNAs between the gefitinib-resistant PC-9 cell line and the gefitinib-sensitive PC-9 cell line by using the microarray technology. Then we analyzed differentially expressed lncRNAs and mRNAs with the cutoff |Fold change| ≥ 2, false discovery rate (FDR) ≤ 0.05, and p-value < 0.05 in the paired gefitinib-resistant and gefitinib-sensitive PC-9 cell lines.

### Functional Enrichment Analysis of DE-mRNAs

Gene Ontology (GO) provides a controlled vocabulary to describe attributes of genes and gene products in any organism from the following three categories: biological processes (BPs), molecular functions (MFs), and cellular components (CCs) (Lovering et al*.*, 2009). Kyoto Encyclopedia of Genes and Genomes (KEGG) is an online database of genomes, enzymatic pathways, biochemistry, diseases, and drugs, allowing us to detect the biological pathway with the significantly enriched mRNAs. Remarkably enriched GO terms and KEGG pathways were determined by p-values <0.05, and their corresponding significance was denoted by the −log10 (p-value) representing the enrichment score.

### Construction of the lncRNA–miRNA–mRNA Regulatory Network Related to Epidermal Growth Factor Receptor Tyrosine Kinase Inhibitor Resistance

We identified the potential microRNA response elements (MREs) of lncRNAs and mRNAs by starBase 2.0, which can provide information for studying microRNAs–ncRNAs, microRNA–mRNA, and RNA–RNA interactions from CLIP-seq data (Li et al*.*, 2014). Then the ceRNA regulatory network was constructed to illustrate interactions between lncRNAs, microRNAs, and mRNAs in EGFR-TKI resistance by Cytoscape.

### Construction of the LUAD-Related Prognostic Risk Model

The TCGA-LUAD data set was downloaded from The Cancer Genome Atlas database (https://www.cancer.gov). A multivariate Cox regression analysis was applied to identify prognostic-related key genes. Four target mRNAs (PPP2CA, PTEN, MAP2K1, and MCL1) were identified as LUAD-related risk signatures, and then, a Prognosis Index (PI) was constructed based on their expression coefficients. According to the PI, LUAD patients were classified into a low-risk group and a high-risk group for the follow-up analysis and study.

## Results

**FIGURE 1:**
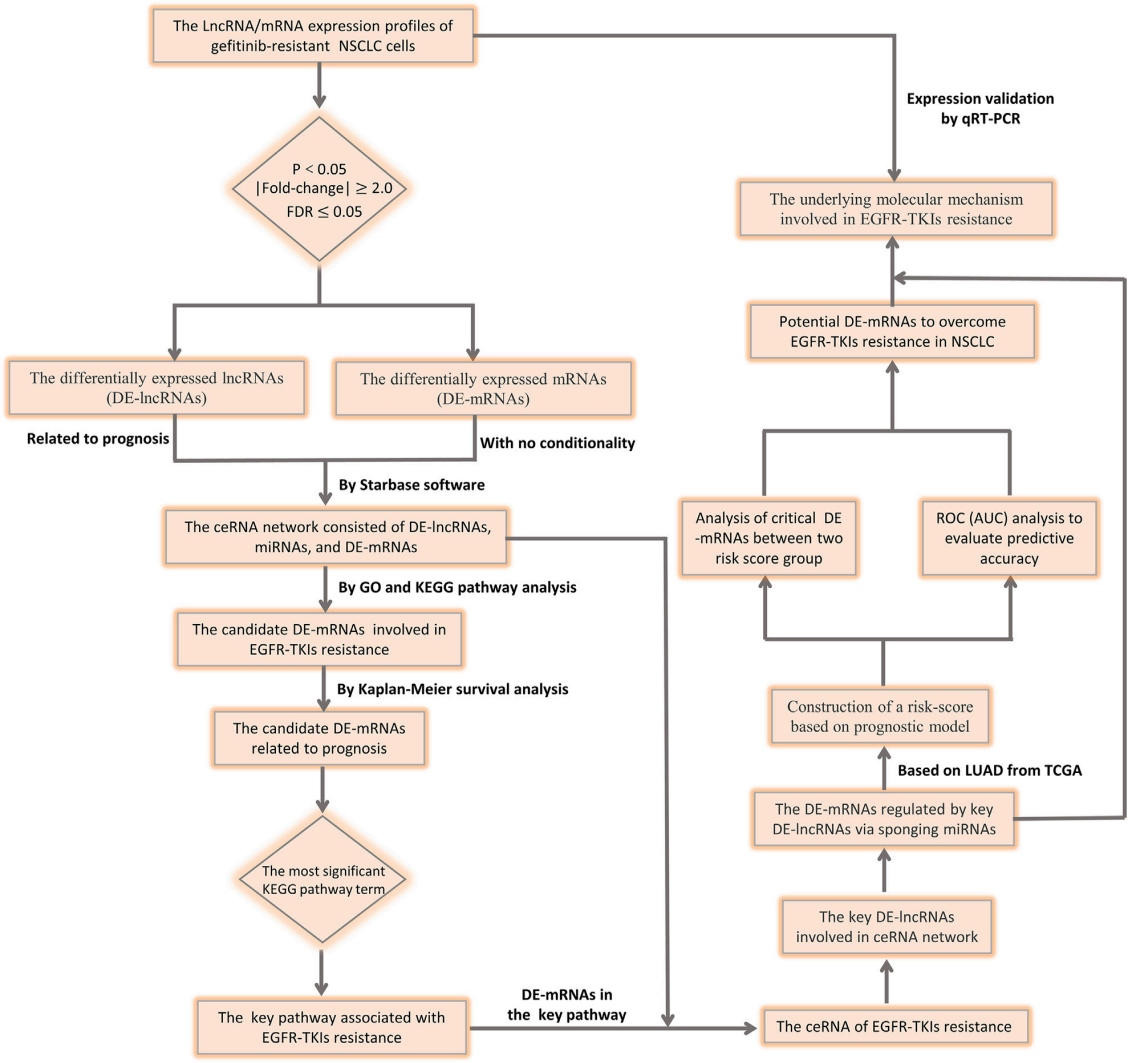
Flow chart of this study design.

Our analysis strategy of this study is shown in a flow chart (Figure 1).

### Model Identification of the Gefitinib-Resistant PC-9 Cell Line

**FIGURE 2:**
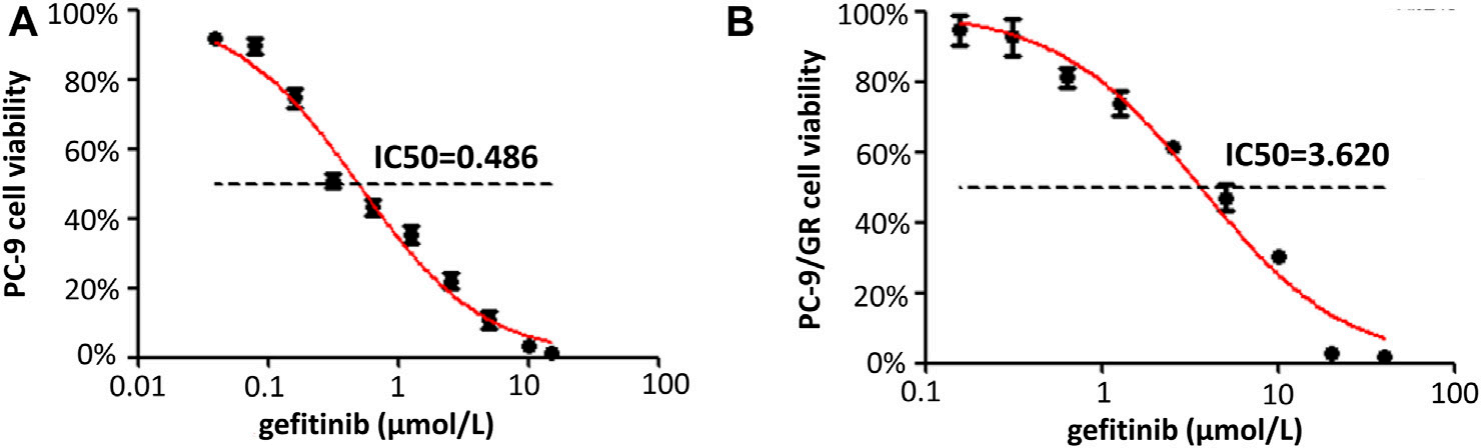
Characterization of the gefitinib-resistant PC-9 cell line. **(A,B)** Growth-inhibitory curves of the two cell lines (PC-9 and PC-9/GR) were determined after 96 h of gefitinib stimulation by the CCK-8 assay (***p < 0.001).

After exposing to a stepwise escalation of the concentration of gefitinib from 0.1 to 15 μM over 8 months, the gefitinib resistance index (RI) was estimated by comparing the half-maximal inhibitory concentration (IC_50_) value of the gefitinib-resistant PC-9 cell line (PC-9/GR) with the paired gefitinib-sensitive PC-9 cell line (PC-9). IC_50_ values of gefitinib-resistant and gefitinib-sensitive PC-9 cell lines after exposing to gefitinib for 96 h were detected by the CCK-8 assay. The CCK-8 assay showed that the IC_50_ value of PC-9/GR for gefitinib was 3.620 ± 0.478 μmol/L, which increased about 7.8-fold compared with that of PC-9 cells (IC_50_: 0.486 ± 0.055 μmol/L) (Figures 2A,B). Distinctly, the gefitinib-resistant PC-9 cell line was more resistant to gefitinib than the gefitinib-sensitive PC-9 cell line, consolidating a solid foundation for this study.

### Functional Enrichment Analysis of Target DE-mRNAs

**FIGURE 3:**
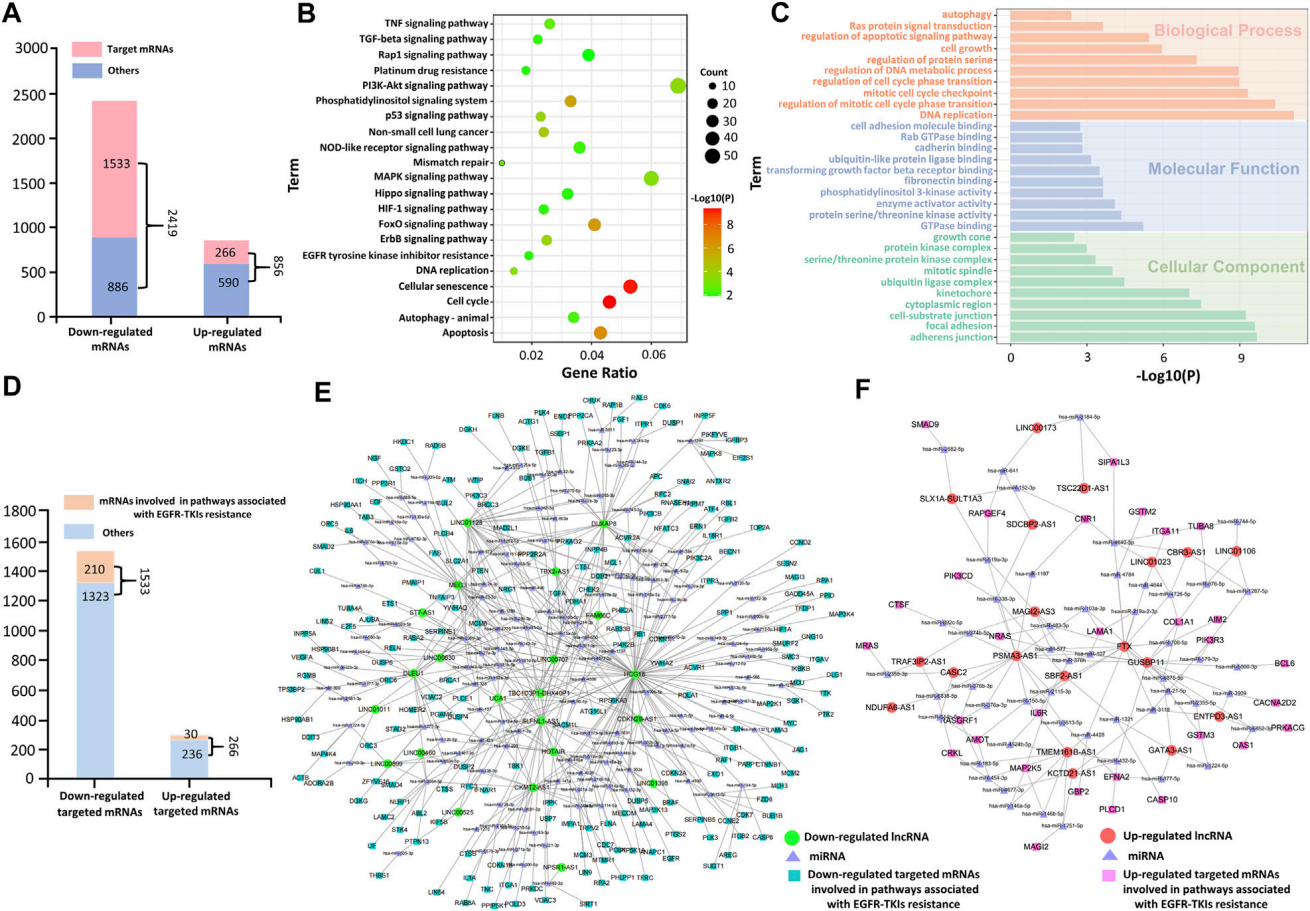
Functional enrichment analysis of the target DE-mRNAs. **(A)** Differentially expressed mRNAs in the PC-9/GR cell line. **(B)** KEGG pathway enrichment analyses of the target DE-mRNAs. **(C)** GO analyses of the target DE-mRNAs. **(D)** Target DE-mRNAs involved in tumor-associated pathways. **(E)** ceRNA network for downregulated DE-mRNAs involved in tumor-associated pathways. **(F)** ceRNA network for upregulated DE-mRNAs involved in tumor-associated pathways.

The 632 DE-lncRNAs and 3275 DE-mRNAs were identified by a microarray analysis. Among 632 DE-lncRNAs, the 23 upregulated lncRNAs and 22 downregulated lncRNAs (Supplementary Figure S1) were significantly associated with the overall survival of LUAD, analyzed by the Kaplan–Meier survival analysis, and selected for the construction of the ceRNA network. As shown in Figure 3A, among the 3275 target DE-mRNAs (2419 downregulated mRNAs and 856 upregulated mRNAs), the starBase 2.0 analysis results indicated that the 1533 downregulated mRNAs and 266 upregulated mRNAs can be regulated by the selected lncRNAs *via* sponging miRNAs, respectively. In most cases, lncRNAs function as members of the ceRNA regulatory network to regulate the expression of target mRNAs and then influencing tumor progression. To explore the potential roles of the selected lncRNAs acting as an endogenous miRNA sponge (ceRNA), GO enrichment and KEGG pathway analyses were performed for the target DE-mRNAs. It can be seen from Figure 3B that the KEGG pathway enrichment analysis showed that the target DE-mRNAs were significantly enriched in pathways involved in EGFR-TKI resistance of NSCLC, such as the PI3K/Akt pathway, MAPK signaling pathway, cell cycle, and apoptosis. The functional enrichment analysis implied that these target DE-mRNAs likely play a role in EGFR-TKI resistance of NSCLC.

In addition, GO functional enrichment analysis showed that the target DE-mRNAs were significantly enriched with biological processes implicated in drug resistance (Figure 3C). The BP enrichment analysis showed that DE-mRNAs were, for the most part, enriched in the regulation of apoptotic signaling pathways. The MF analysis predicted a close association between DE-mRNAs and the protein serine/threonine kinase activity, enzyme activator activity, and phosphatidylinositol 3-kinase activity. The CC enrichment analysis denoted that DE-mRNAs were mainly enriched in the cytoplasmic region.

In Figure 3D, 1799 target DE-mRNAs included 1533 downregulated mRNAs and 266 upregulated mRNAs, of which 240 target DE-mRNAs (210 downregulated mRNAs and 30 upregulated mRNAs) were mapped into 21 tumor-associated pathways, as shown in Figure 3B. To further clarify their roles in the ceRNA network, the ceRNA regulatory networks based on these mRNAs are presented in Figures 3E,F. Results show that 210 downregulated mRNAs and 30 upregulated mRNAs were regulated by the 22 selected downregulated lncRNAs and 19 upregulated lncRNAs *via* sponging miRNAs, respectively, which indicates that these mRNAs are the key regulatory genes of the selected DE-lncRNAs. Therefore, the selected DE-lncRNAs play crucial roles in EGFR-TKI resistance by regulating the target DE-mRNAs involved in tumor-associated pathways.

### Revealing the Potential Genes Associated With Epidermal Growth Factor Receptor Tyrosine Kinase Inhibitor Resistance Among Target DE-mRNAs

**FIGURE 4:**
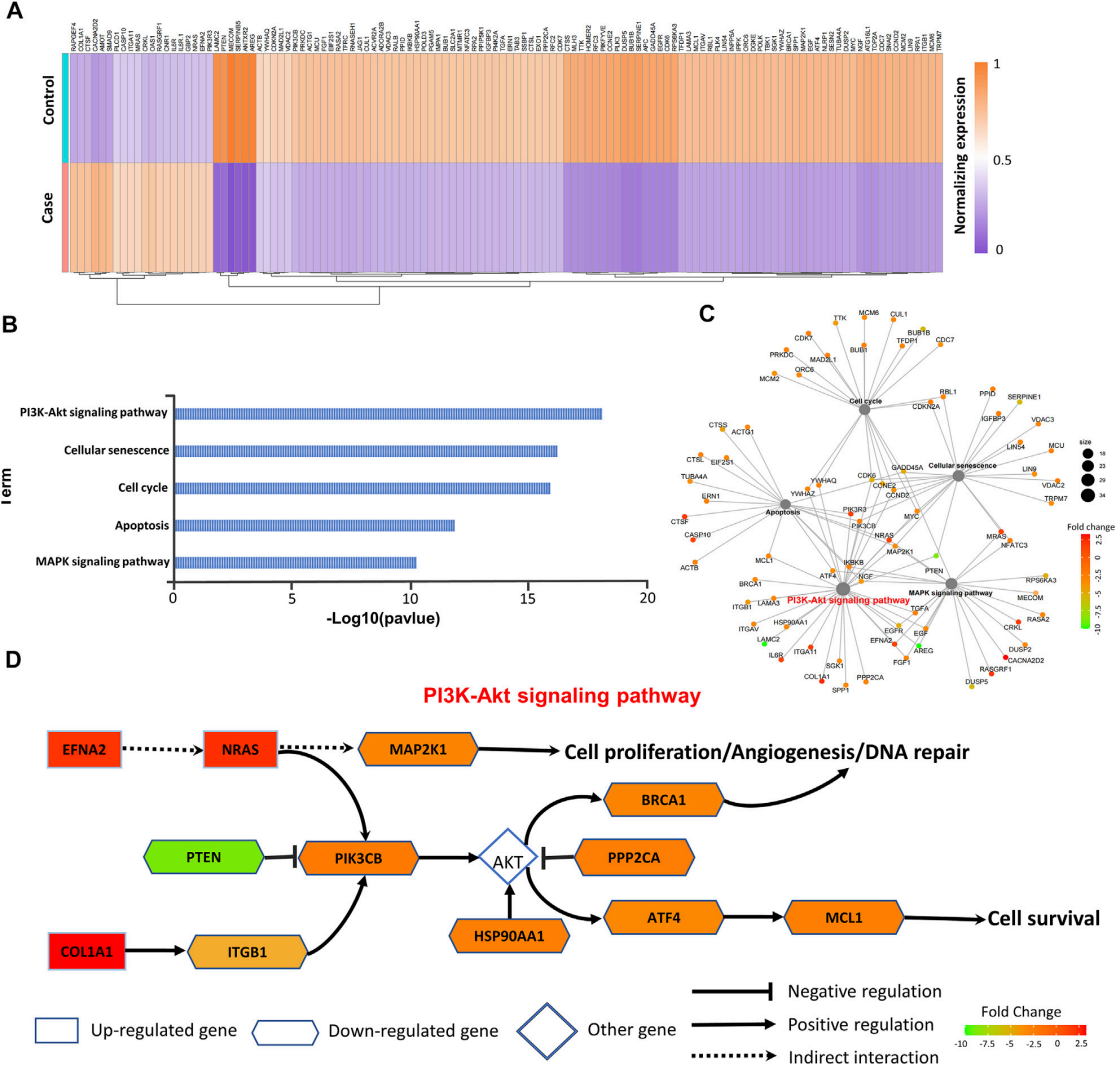
Analysis of prognosis-related mRNAs. **(A)** In total, 122 prognosis-related mRNAs are shown in heat map. **(B,C)** KEGG pathway enrichment analyses of 122 prognosis-related mRNAs. **(D)** Underlying mechanism of drug resistance to EGFR-TKIs revealed by target DE-mRNAs.

A prognostic analysis was carried out to explore the prognostic significance of these 240 target DE-mRNAs involved in EGFR-TKI resistance. Based on The Cancer Genome Atlas (TCGA) analysis, we had identified 122 prognosis-related target DE-mRNAs (Supplementary Figure S2). These 122 prognosis-related mRNAs not only represented significant changes in the gene expression (Figure 4A) but also were most enriched in the PI3K/Akt pathway closely related to EGFR-TKI resistance (Figures 4B,C). Figure 4D shows the underlying mechanism revealed by the functional analysis on the prognosis-related target DE-mRNAs. It can be seen from Figure 4D that the downregulation of PTEN would alleviate the suppression of PIK3CB and further activate AKT together with the downregulation of PPP2CA, which may cause various biological processes including cell proliferation, angiogenesis, and DNA repair, inferring the signal response caused by PTEN would activate the PI3K/Akt signaling pathway, resulting in a close relationship with the development of drug resistance to EGFR-TKIs.

### Underlying ceRNA Regulatory Mechanism of Epidermal Growth Factor Receptor Tyrosine Kinase Inhibitor Resistance

**FIGURE 5:**
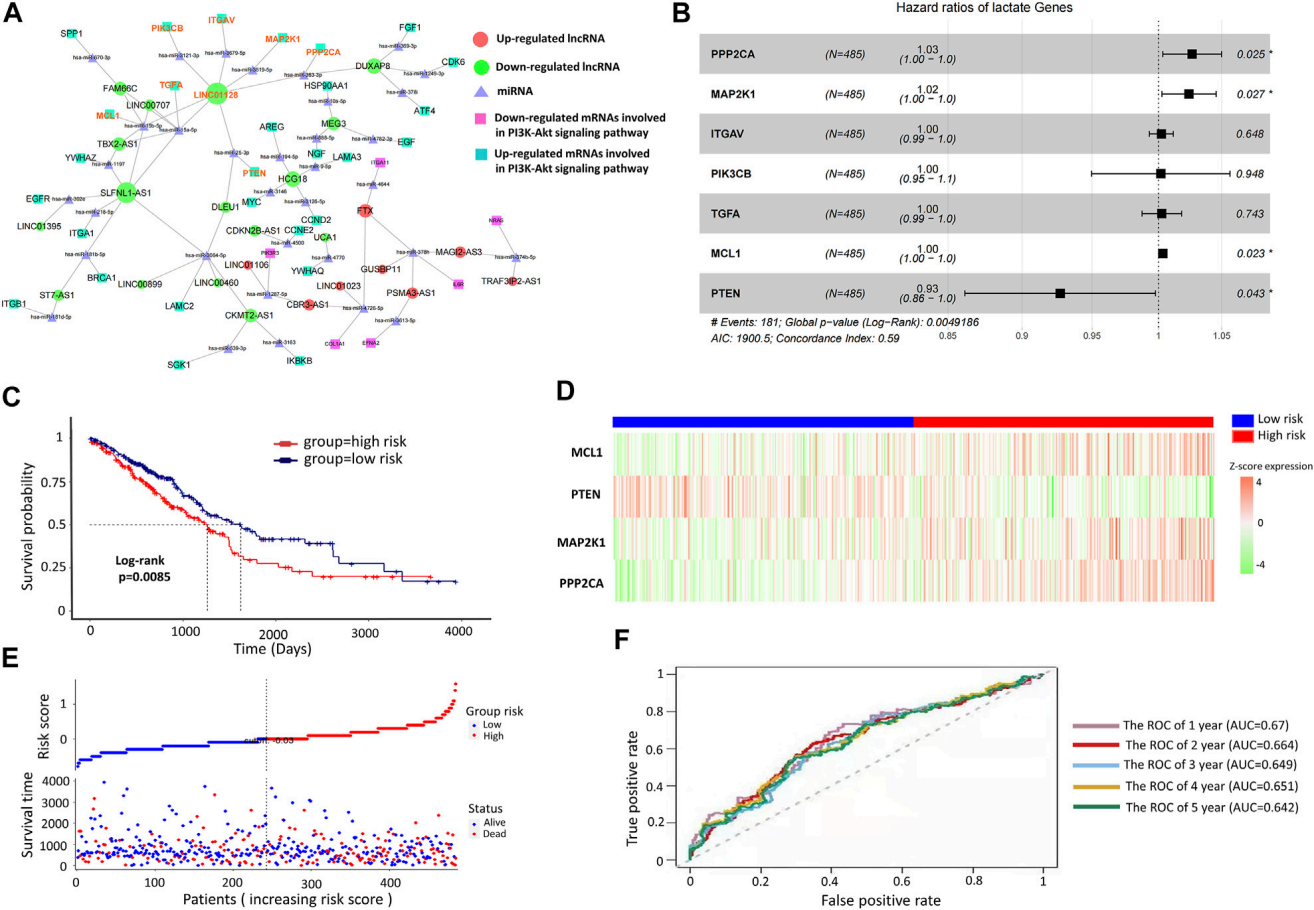
Revealing LUAD-related risk signature genes based on the risk model. **(A)** ceRNA for prognosis-related mRNA involved in the PI3K/Akt signaling pathway. **(B)** Univariate Cox regression analysis of the seven DE-mRNAs regulated by LINC01128. **(C)** Low-risk group had a longer OS than the high-risk group. **(D)** Expression levels of four signature genes of patients in the low-risk group and high-risk group. **(E)** Number of patients in the two groups is ranked by the risk score and the survival time of patients in the TCGA data set, respectively. **(F)** ROC analysis of the LUAD-related risk signature genes in the first 5 years.

To understand how the selected DE-lncRNAs regulate the mRNAs involved in the PI3K/Akt signaling pathway, the lncRNA–miRNA–mRNA network is constructed for them (Figure 5A). As shown in Figure 5A, LINC01128 regulates most of the genes involved in the PI3K pathway, including PTEN (the key negative regulator) *via* sponging miRNAs, and thus it serves as a key lncRNA involved in EGFR-TKI resistance. Then based on the LUAD data set from TCGA, we explored the roles of these mRNAs regulated by LINC01128 as a risk signature to predict the prognosis of LUAD. As shown in Figure 5B, a multivariate Cox regression analysis was performed according to these seven DE-mRNAs regulated by LINC01128. According to the multivariate Cox regression analysis, the weighted coefficients of the four mRNAs (PPP2CA, PTEN, MAP2K1, and MCL1) with significant difference (p < 0.05) were obtained and used to construct the following Prognosis Index (PI) formula: PI= (0.192 × PPP2CA expression value) + (−0.561 × PTEN expression value) + (0.175 × MAP2K1 expression value) + (0.025 × MCL1 expression value). In the risk model, the patients from the TCGA-LUAD data set were grouped into high-risk (*n* = 218) and a low-risk groups (*n* = 267) according to the median risk score. The survival difference between the two groups was indicated by the Kaplan–Meier (log-rank) survival analysis. As shown in Figure 5C, the overall survival (OS) of LUAD patients with high risk (red line) was significantly (p = 0.0085) shorter than that with low risk (blue line). In addition, the expression of these four mRNAs had changed significantly between the high-risk and low-risk groups (Figure 5D). Moreover, as shown in Figure 5E, a rising curve could be obtained by sorting the risk scores of patients and dividing them into two groups, where the number of deaths in the high-risk group was higher than that in the low-risk group. The predictive effect of PI for the first 5 years was evaluated by the ROC curve (Figure 5F). In summary, our results provided key DE-mRNAs regulated by LINC01128 as a risk signature for EGFR-TKI treatment of NSCLC.

**FIGURE 6:**
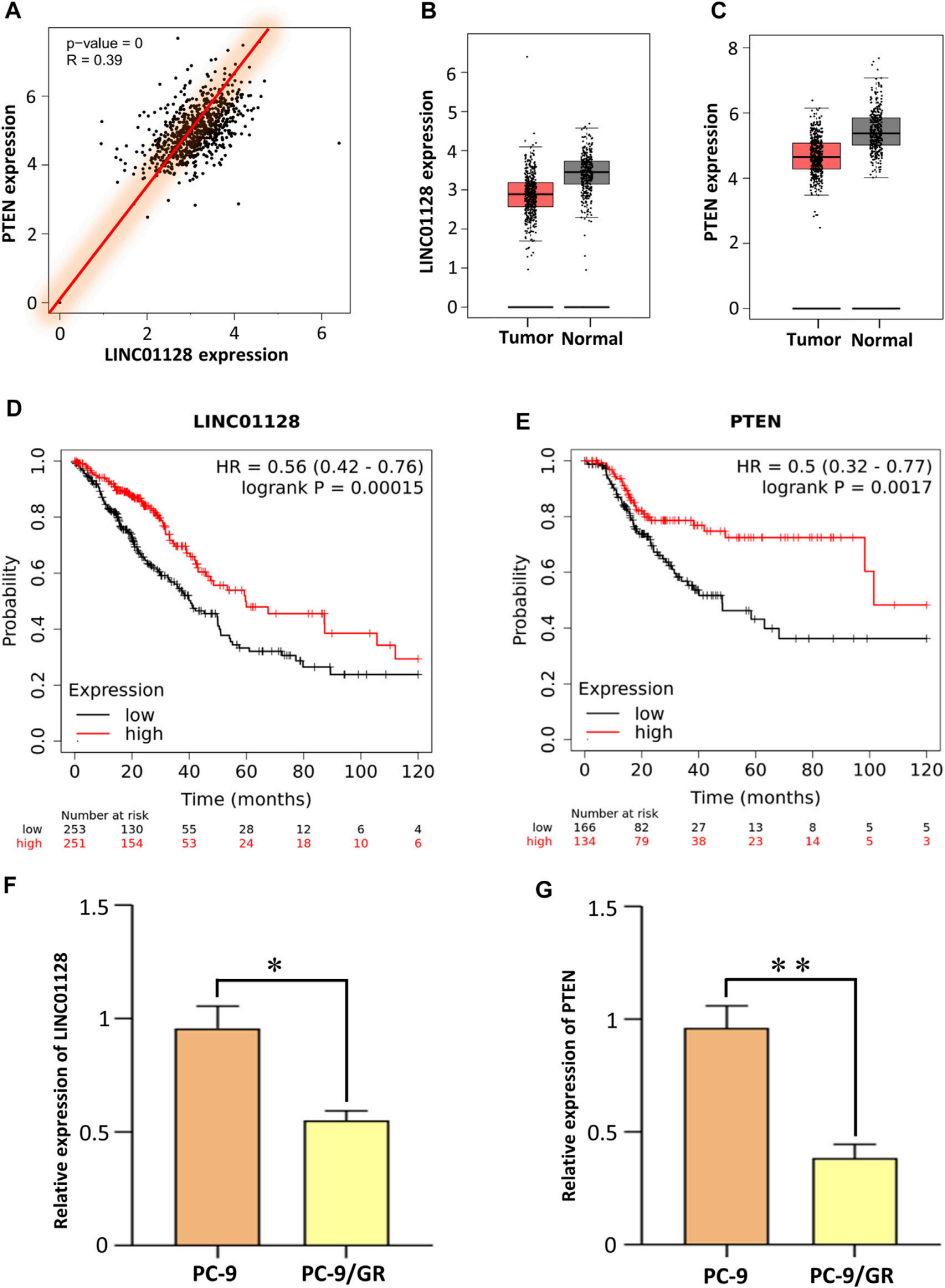
Analysis of the gene expression and qRT-PCR validation for LINC01128 and PTEN. **(A)** Correlation between LINC01128 and PTEN. **(B,C)** Gene expression levels of LINC01128 and PTEN in tumor and normal tissues, respectively. **(D,E)** Kaplan–Meier survival curve of LINC01128 and PTEN. **(F,G)** Expression level of LINC01128 and PTEN was assessed by the qRT-PCR analysis (*p < 0.05 and **p < 0.01).

To further confirm the possible interaction of LINC01128 and PTEN, a correlation analysis based on TCGA data was carried out between their expression levels. It is shown in Figure 6A that the correlation between LINC01128 and PTEN shows a positive linear relationship with Pearson’s correlation coefficient of R = 0.39 and p-value = 0. In addition, their expression levels were decreased in the tumor tissue compared to non-tumor tissue (Figures 6B,C). Moreover, the survival analysis consistently revealed that the low expression of these two genes was associated with poor survival (Figures 6D,E). In addition, as shown in Supplementary Table S1, the reduction in the expression of LINC01128 and PTEN was detected in several EGFR-TKI–resistant data sets (GSE71587, GSE33658, and GSE23206) obtained from the GEO database (https://www.ncbi.nlm.nih.gov/geo/). Therefore, according to the ceRNA regulatory mechanism, we considered that LINC01128 downregulated PTEN by competitively sponging miR-25-3p to promote EGFR-TKI resistance *via* the PI3K/Akt signaling pathway in NSCLC.

To validate the gene expression profiles of LINC01128 and PTEN, their expression levels were assessed by a qRT*-*PCR analysis. The results showed that the change in trends of these two genes was consistent with the microarray data (Figures 6F,G), suggesting that our studies were highly reliable.

## Discussion

Drug resistance to EGFR-TKI treatments is the main cause of clinical treatment failure in NSCLC. There have been numerous studies on the mechanism of EGFR-TKI resistance, including the T790M mutation, ERBB2 amplification, MET alterations, SCLC transformation, and so forth (Oxnard et al*.*, 2018; Piper-Vallillo et al., 2020). However, the underlying molecular mechanisms involved in resistance to EGFR-TKIs in NSCLC remain to be elucidated. Numerous lncRNAs have been proved to contribute to the drug resistance of NSCLC (Liu et al*.*, 2020; Wang Z. et al., 2021). In recent years, a comprehensive research of differential expression profiles of lncRNAs and their related ceRNA networks involved in drug resistance of NSCLC has drawn more and more attention. For instance, lncRNA CASC9 repressed the DUSP1 expression to promote EGFR-TKI resistance by recruiting the histone modification enzyme (Chen Z. et al., 2020). Wu et al. reported that lncRNA APCDD1L-AS1 acted as a miRNA sponge by targeting miR-1322/miR-1972/miR-324-3p and subsequently removed their transcription inhibition of SIRT5, leading to elevated expression of EGFR and enhanced EGFR-TKI resistance (Wu et al., 2021). Recently, Wang et al. found that two lncRNAs, ABTB1 and NPTN, exerted their functional roles *via* targeting the hsa-miR-150–5p/SERPINE1 axis, leading to drug resistance to EGFR-TKIs and an unfavorable survival rate in NSCLC (Wang T. et al., 2021). Nevertheless, little is known about the detailed underlying molecular mechanisms of how the RNA regulatory network participates in mediating EGFR-TKI resistance in NSCLC.

In this study, the DE-lncRNAs and DE-mRNAs were obtained from gefitinib-resistant NSCLC cells using the microarray technology and then used to construct the ceRNA regulatory network for exploring the underlying mechanisms of EGFR-TKI resistance in NSCLC. Our results revealed that the downregulated LINC01128 could decrease PTEN *via* sponging miR-25-3p, and then the signal response caused by the downregulation of PTEN would activate the PI3K/Akt pathway, which may lead to the development of EGFR-TKI resistance in NSCLC. Specifically, the reduction of PTEN subsequently led to activation of its downstream target PI3KCB, which is indispensable for the regulation of various biological processes, including PI3K pathway activation, cell growth, and cell survival (Wee et al., 2008). Our work supports previous studies that the suppression of PTEN plays a very significant role in drug resistance of NSCLC *via* activating the PI3K/Akt signaling pathway (Sun et al., 2019; Xiong et al., 2021). Therefore, the downregulated PTEN activates the PI3K/Akt signaling pathway and may lead to the development of drug resistance to EGFR-TKIs in NSCLC.

To further analyze the underlying mechanism of LINC01128 in PTEN regulation, Pearson’s correlation and survival analyses were performed for the LINC01128 and PTEN. The analyses’ results indicated a correlation between LINC01128 and PTEN, thus showing a positive linear relationship, and their low expression levels were associated with poor prognosis in lung adenocarcinoma (LUAD). In addition, the qRT-PCR analysis indicated that the change in trends of these two genes was consistent with the microarray data, suggesting that our results were relatively reliable. Until now, several ceRNA regulatory networks involved in LINC01128 have been reported. For instance, LINC01128 facilitates cervical cancer by functioning as a sponge of miR-383-5p, thus enhancing the SFN expression and promoting cell proliferation, migration, and invasion (Hu et al., 2019). Li et al. confirmed that the low LINC01128 level could enhance the NR3C2 expression by sequestering miR-4260, resulting in resisting acute myeloid leukemia (Li et al., 2018). Although the roles of LINC01128 in the comprehensive ceRNA network involved in EGFR-TKI resistance in NSCLC are poorly characterized yet, these previous studies suggested that it was essential for cell proliferation, apoptosis, and drug resistance by regulating the expression of corresponding target mRNAs. Based on the above mentioned results, it was inferred that the reduction of PTEN regulated by LINC01128 in our study may promote EGFR-TKI resistance *via* the PI3K/Akt signaling pathway in NSCLC.

The next-generation sequencing (NGS) implementation in a clinical setting may be a suitable solution to validate our proposed molecular mechanism. The adoption of NGS has been reported to save personnel time dedicated to testing activities and to reduce the overall cost of testing per patient (Pisapia et al., 2022). In addition, compared with the real-time polymerase chain reaction (RT-PCR), the adoption of NGS platforms for molecular predictive pathology allows a multiplexing analysis. Although tissue availability of advanced-stage NSCLC patients may limit the application of NGS technology in routine practice for identifying potentially predictive biomarkers, liquid biopsy was treated as a complementary tool to tissue sample molecular testing and may overcome this limitation (Leighl et al., 2019; Russo et al., 2021). Moreover, in contrast to singleplex methods (RT-PCR), the NGS-based techniques can make liquid biopsies more feasible to discover new and reliable biomarkers and achieve early diagnosis of drug resistance in lung cancer (Frenel et al., 2015). Therefore, with the development of liquid biopsy and NGS technology, the NGS-based strategy may help uncover the mechanisms of drug resistance in lung cancer and discover novel therapeutic targets.

There are several advantages in our study. First, it is the first time that we have proposed LINC01128/miR-25-3p/PTEN as a potential ceRNA regulatory axis to promote EGFR-TKI resistance *via* the PI3K/Akt pathway, which provides novel insights into the underlying molecular mechanisms of drug resistance to EGFR-TKIs in NSCLC. Second, our results provide some novel targets regulated by key lncRNAs as risk signatures for EGFR-TKI treatment of NSCLC. In addition, our studies will help find some novel targets to overcome EGFR-TKI resistance in NSCLC.

## Conclusion

In conclusion, our results suggest that LINC01128/miR-25-3p/PTEN may promote EGFR-TKI resistance *via* the PI3K/Akt signaling pathway, which helps in elucidating the underlying molecular mechanisms of EGFR-TKI resistance in NSCLC. In addition, our work will help find some novel drug targets for overcoming resistance to EGFR-TKIs in NSCLC.

## Data Availability

The original contributions presented in the study are publicly available. This data can be found at: GSE193628.
