# Supplementary figures and images for: Analysis of the lncRNA–miRNA–mRNA Network Reveals a Potential Regulatory Mechanism of EGFR-TKI Resistance in NSCLC
Source: Front Genet. 2022 Apr 29;13:851391. doi: 10.3389/fgene.2022.851391 (PMC9099042; doi:10.3389/fgene.2022.851391)

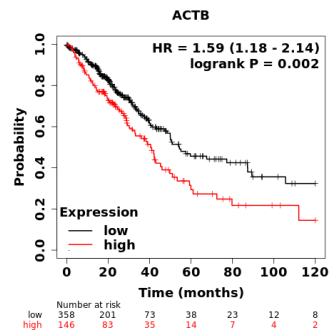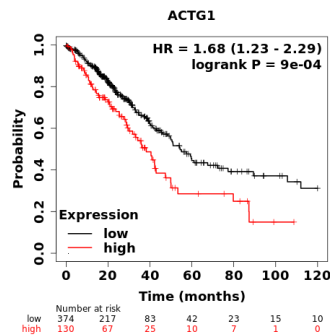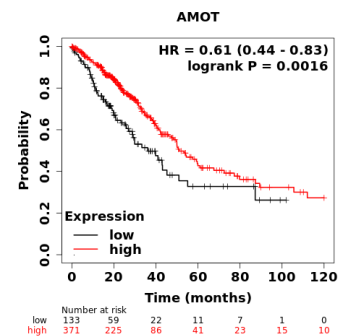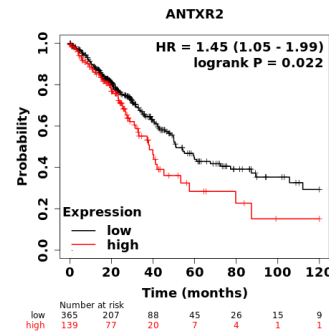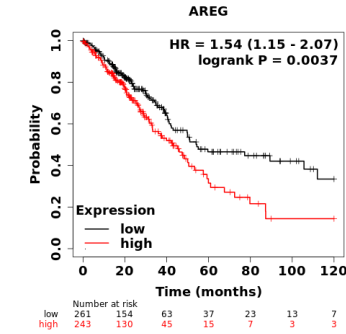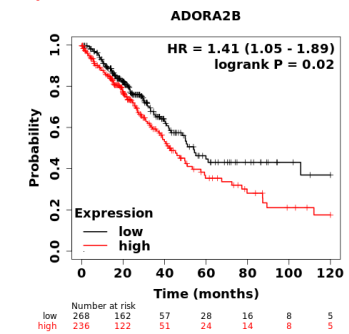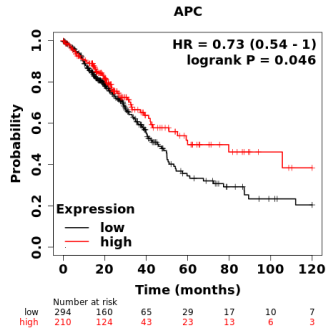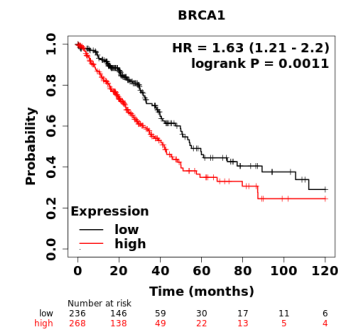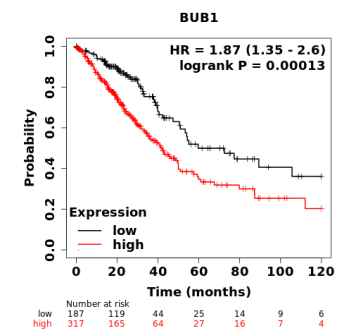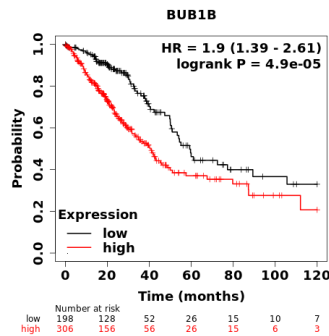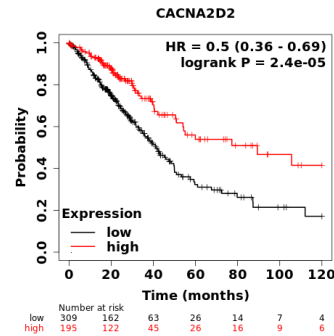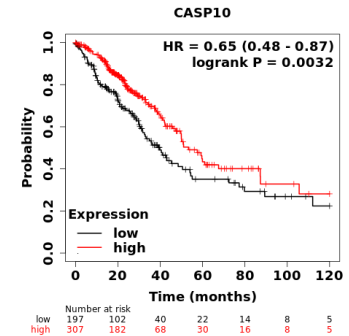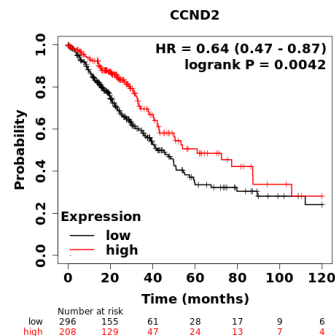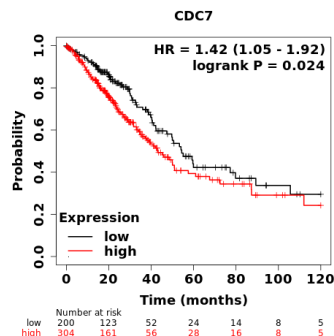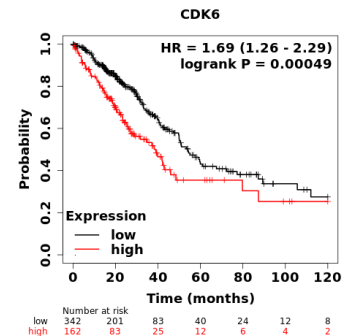

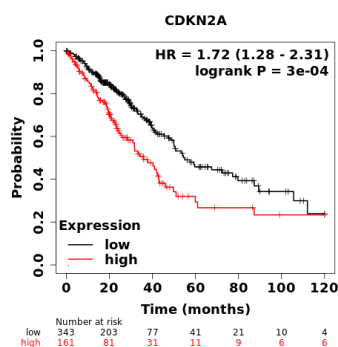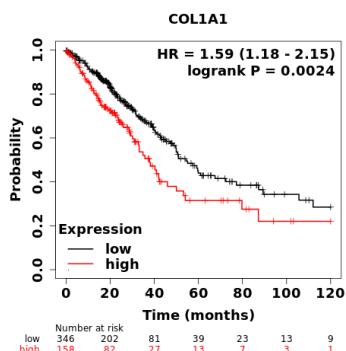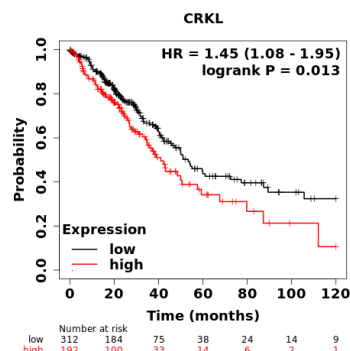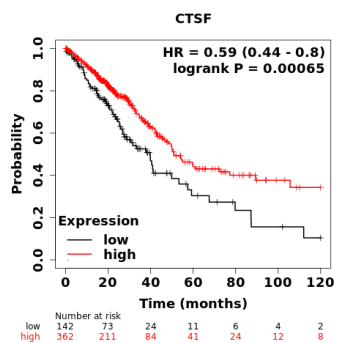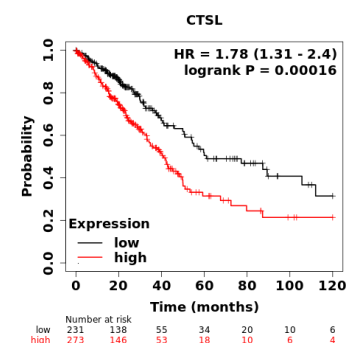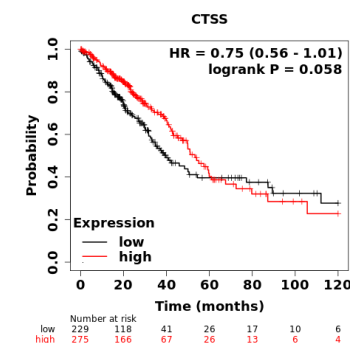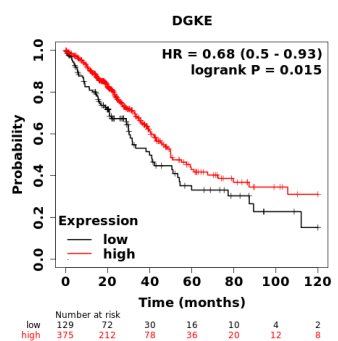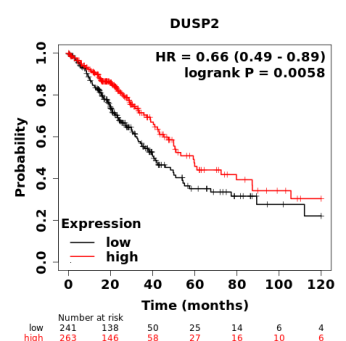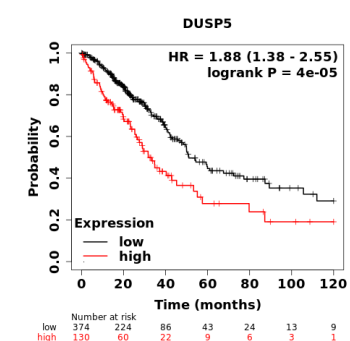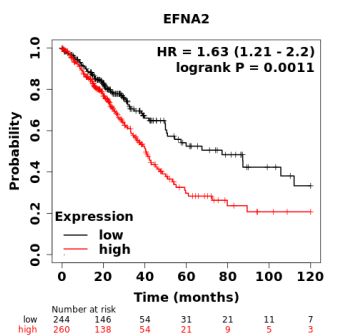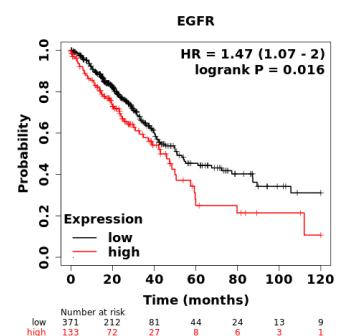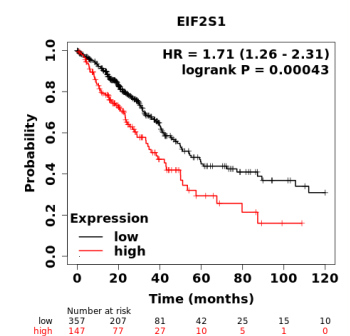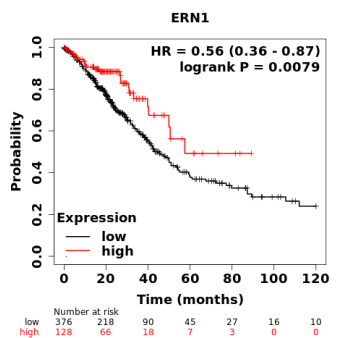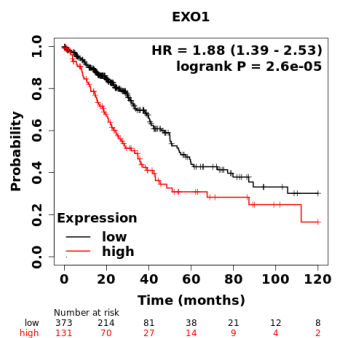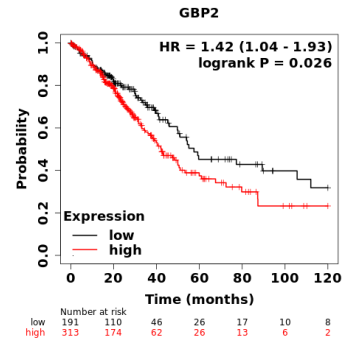

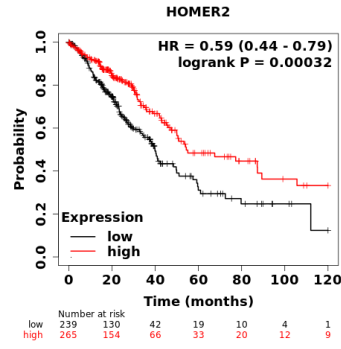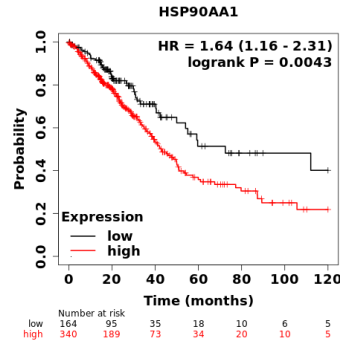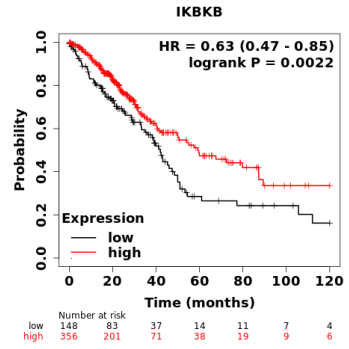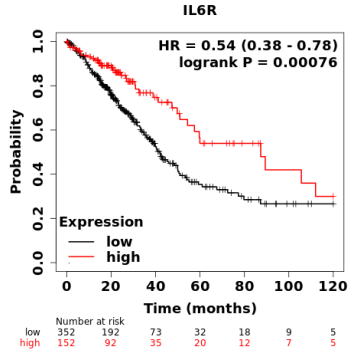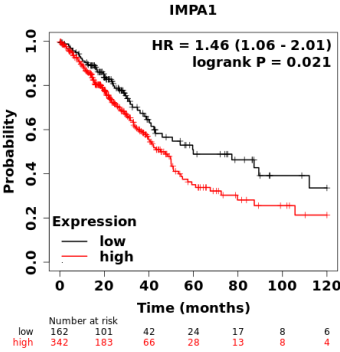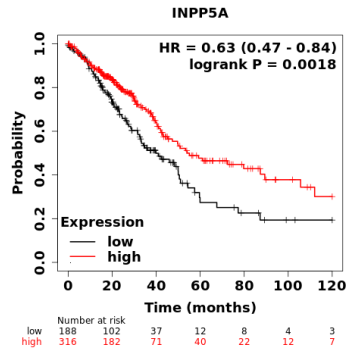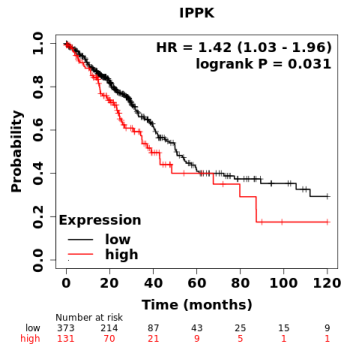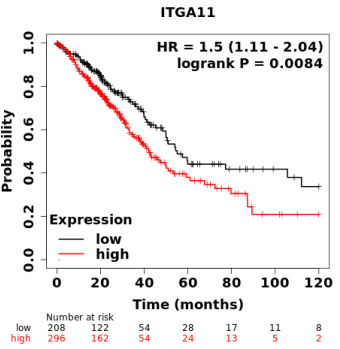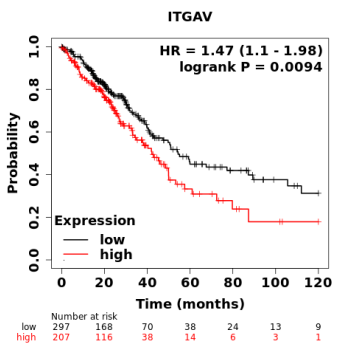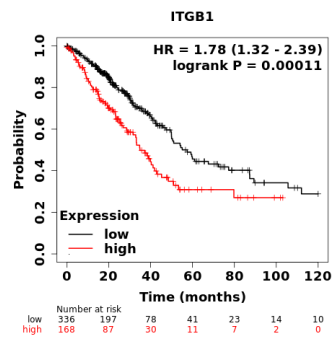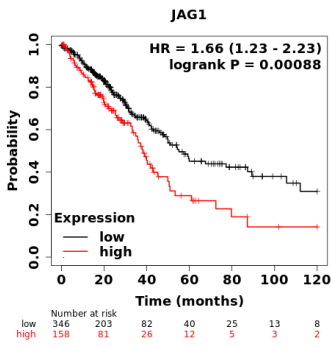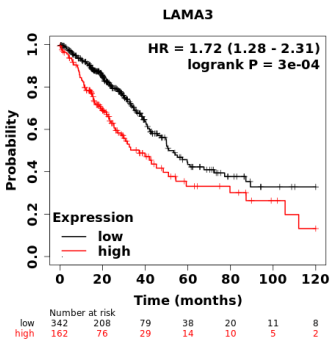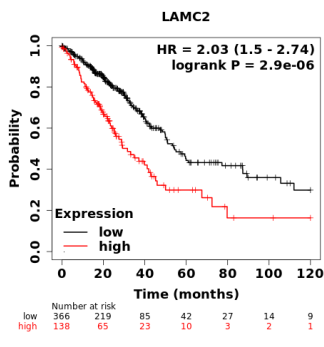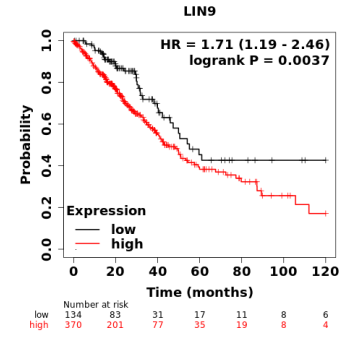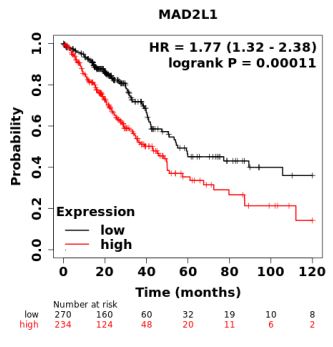

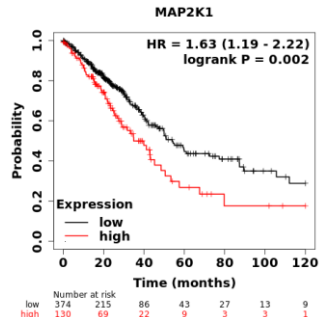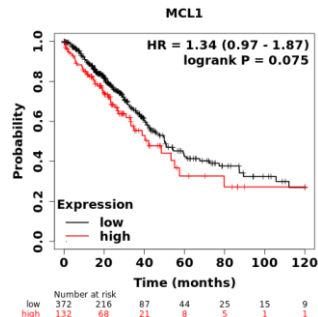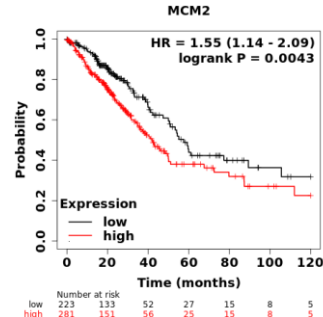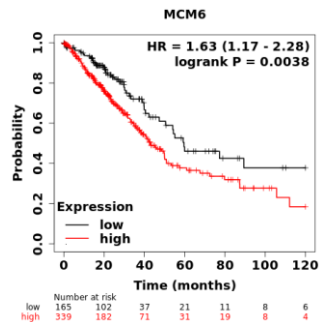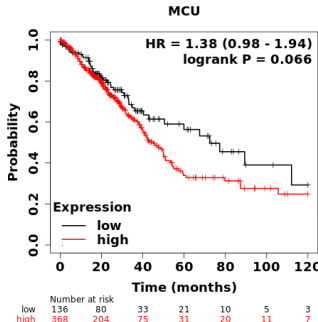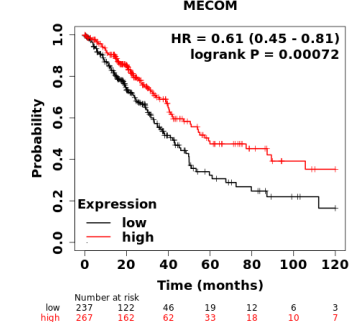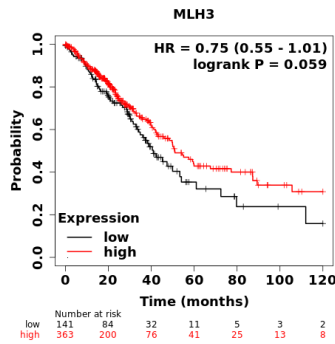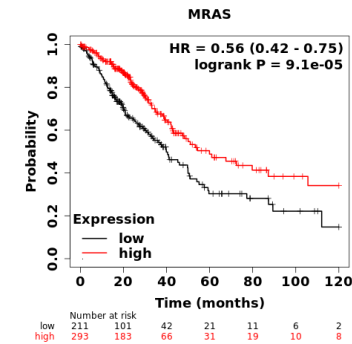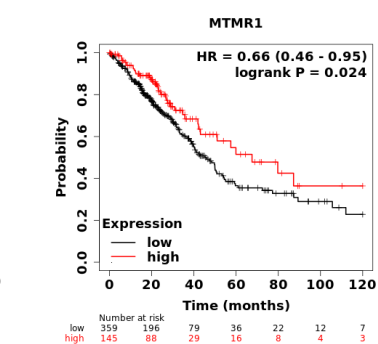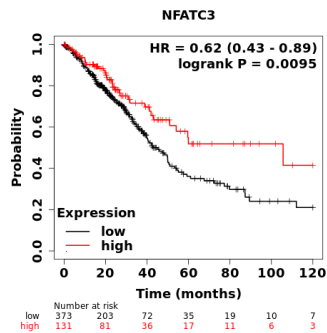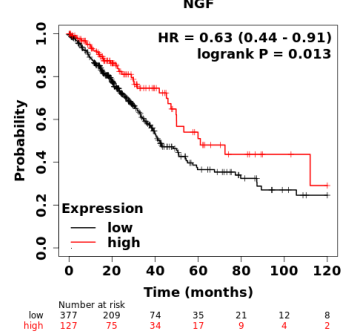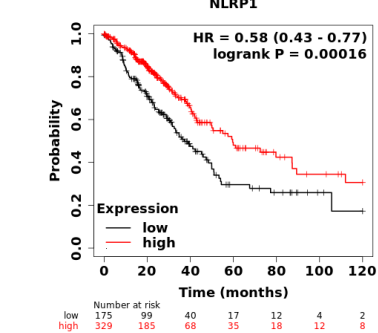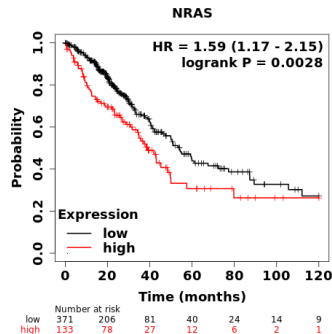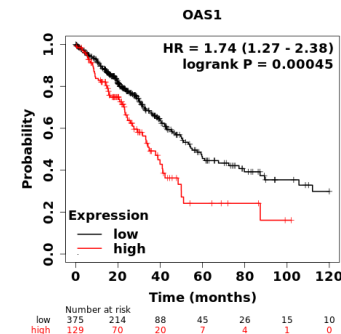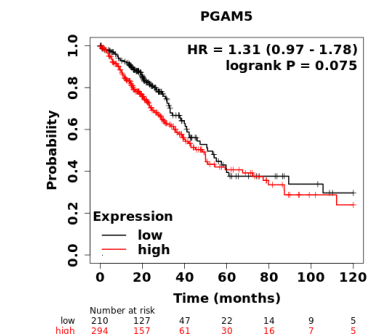

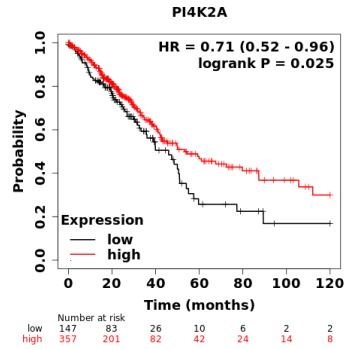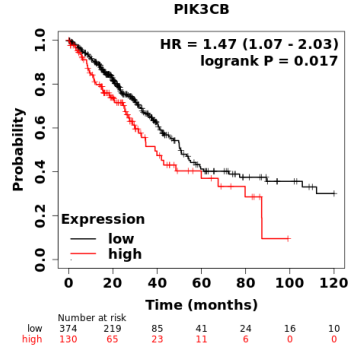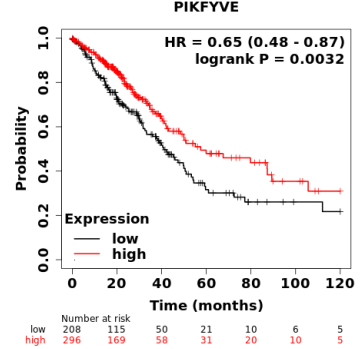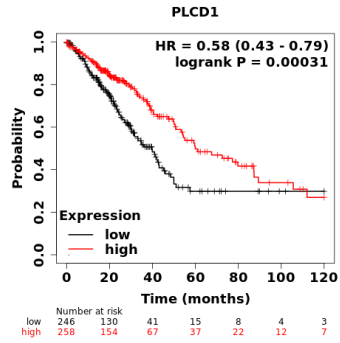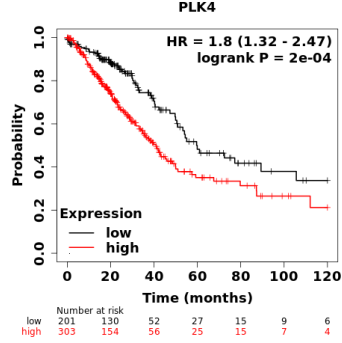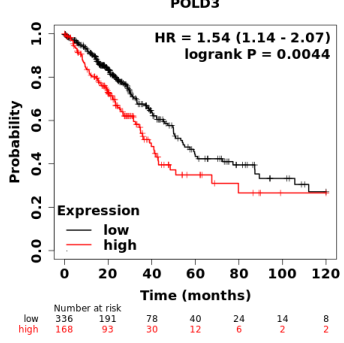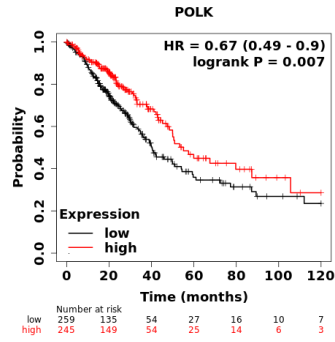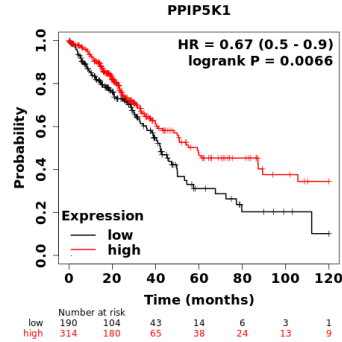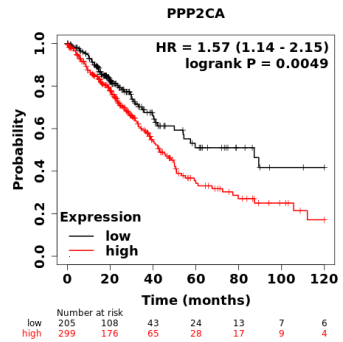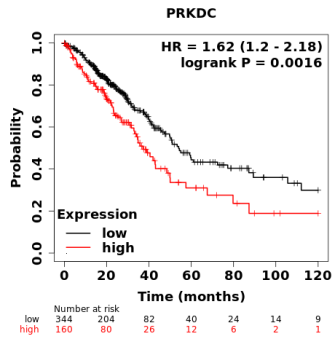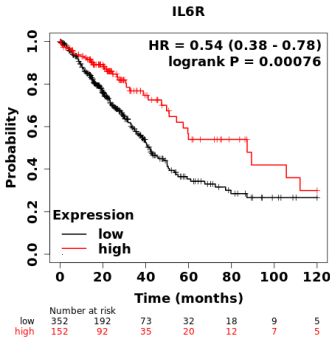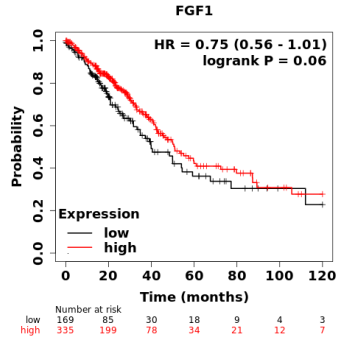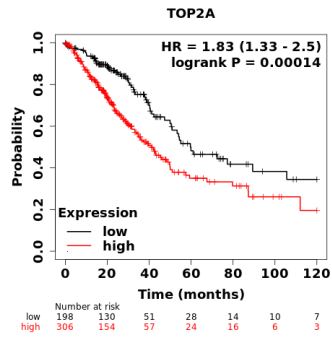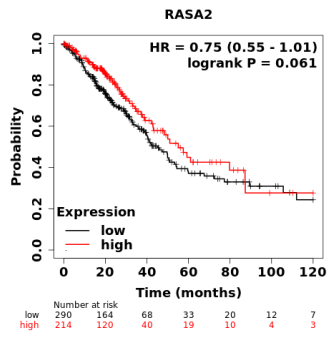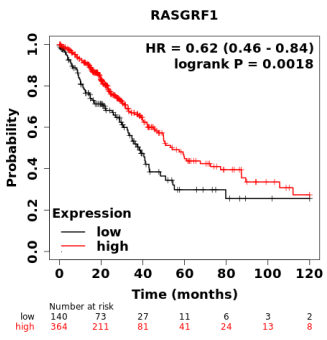

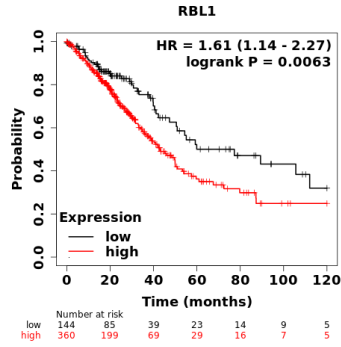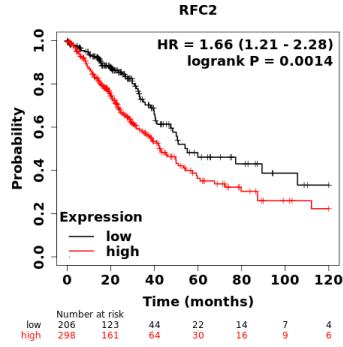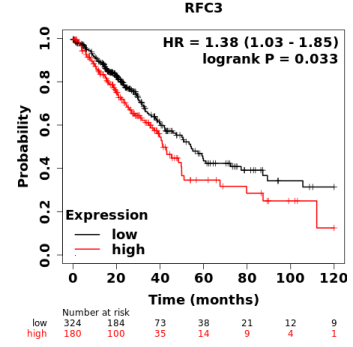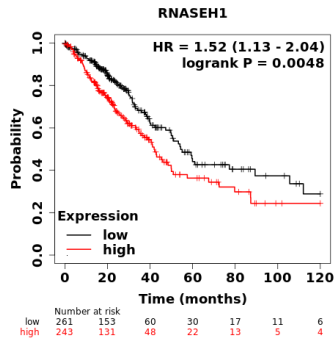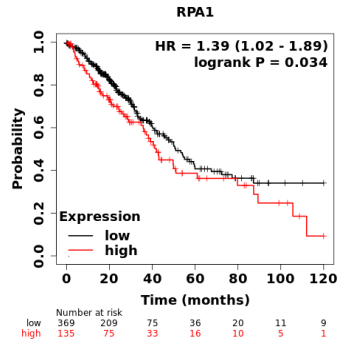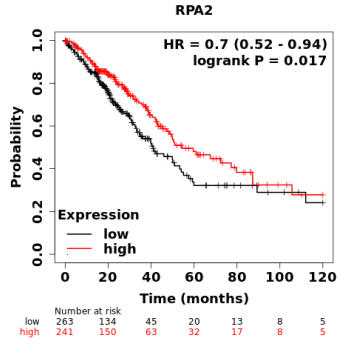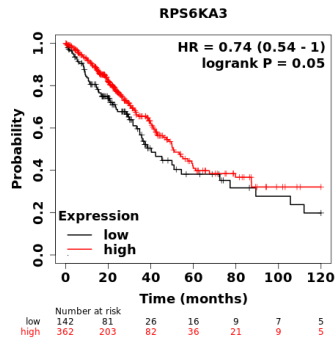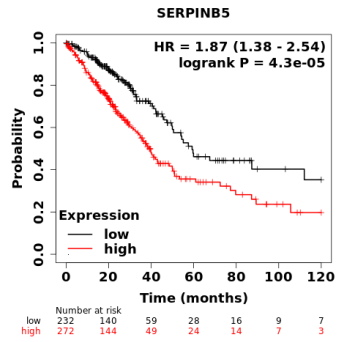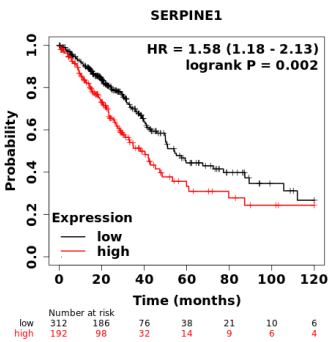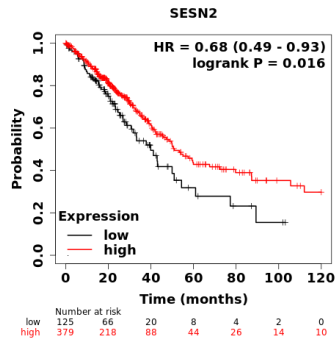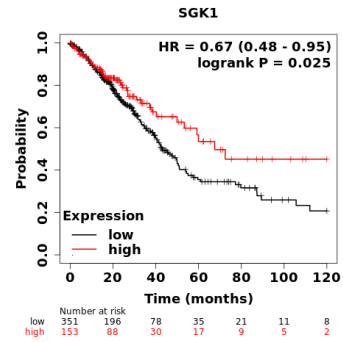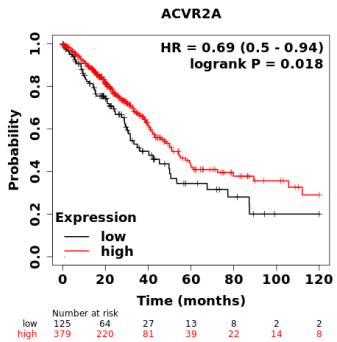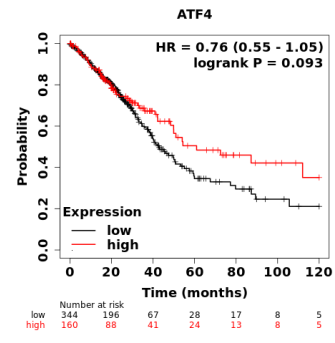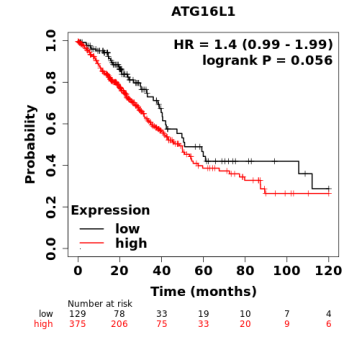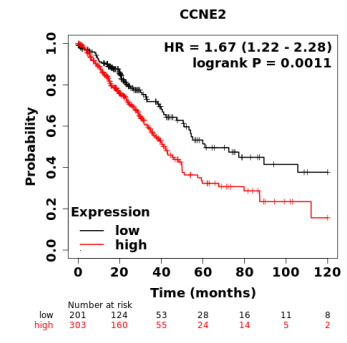

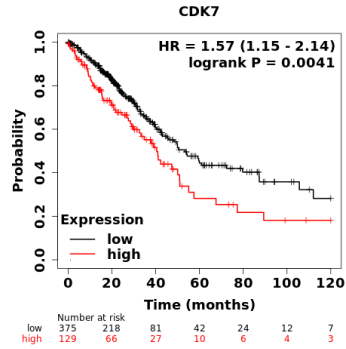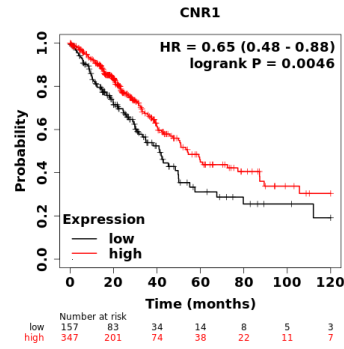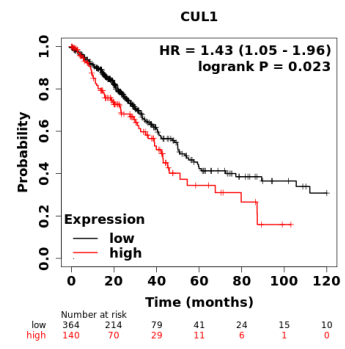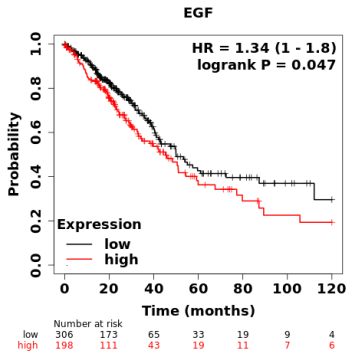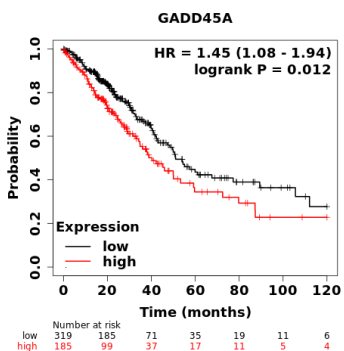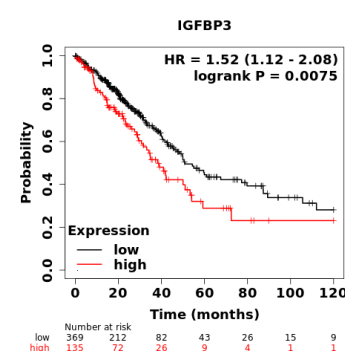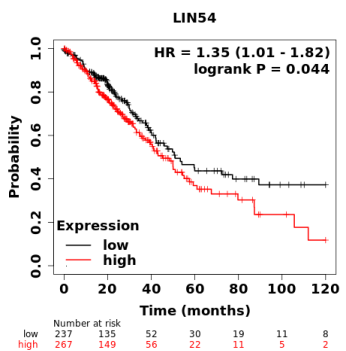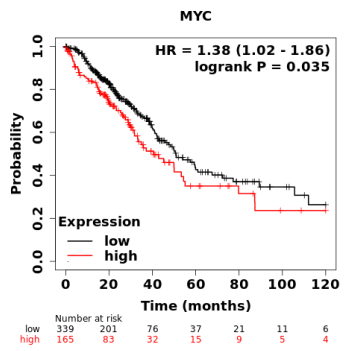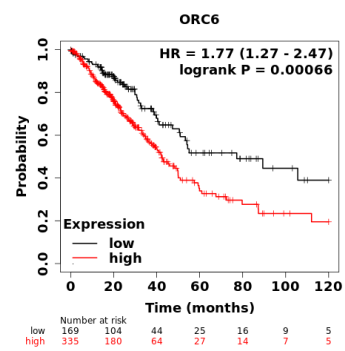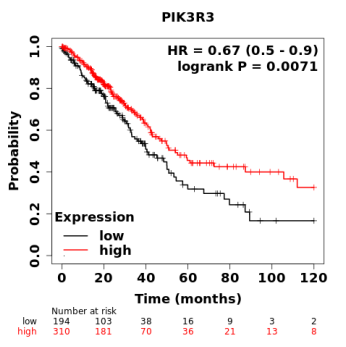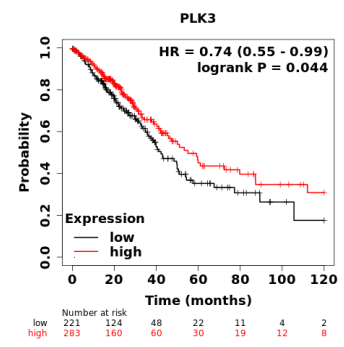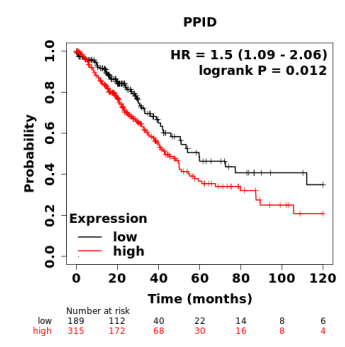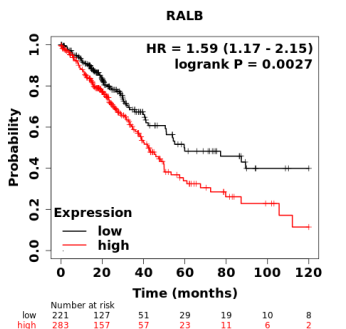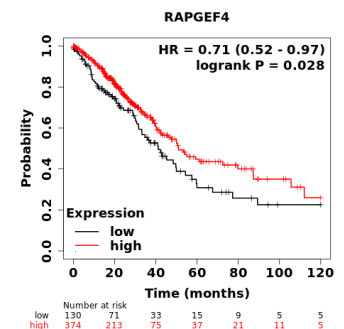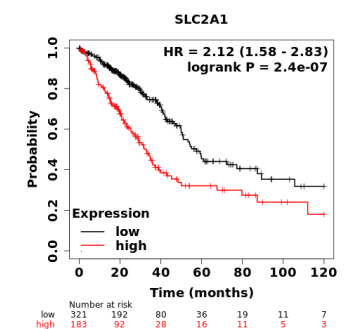

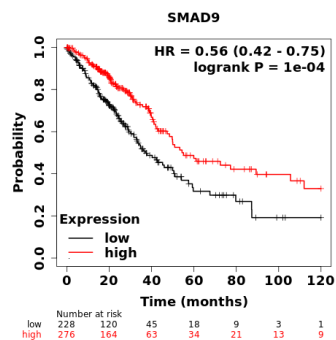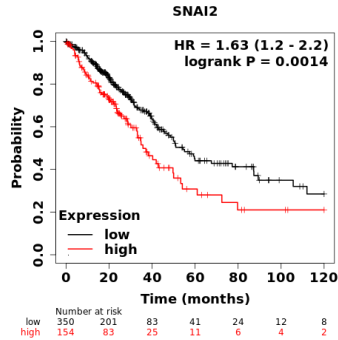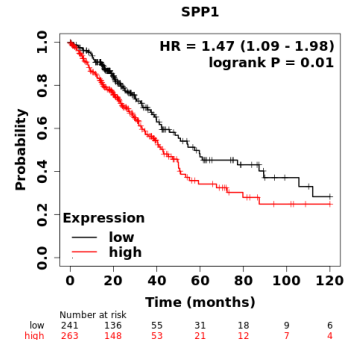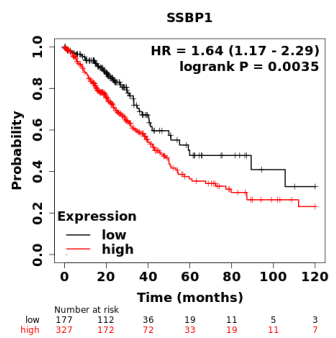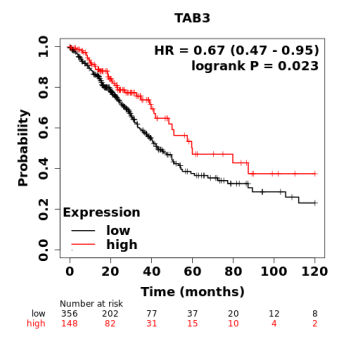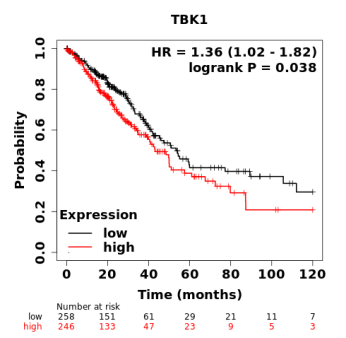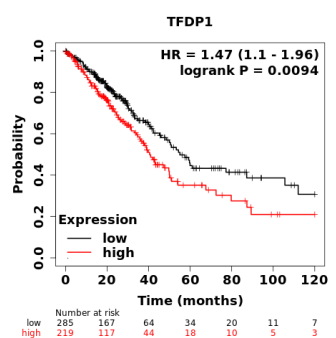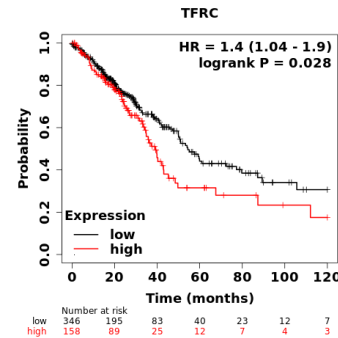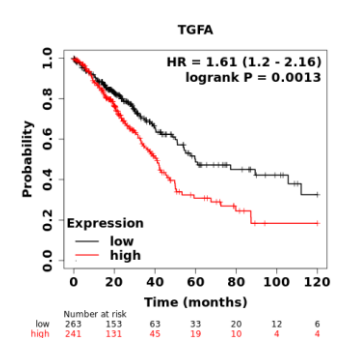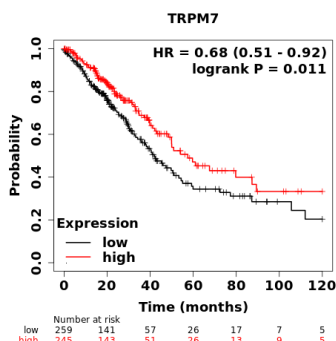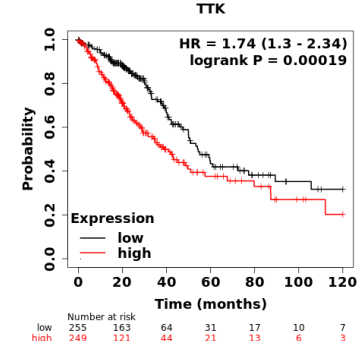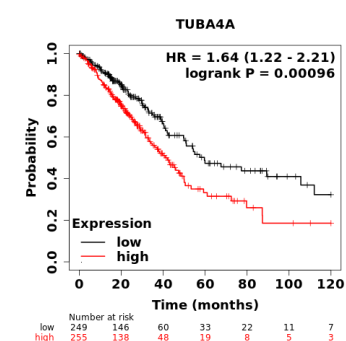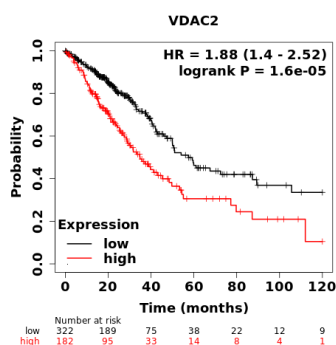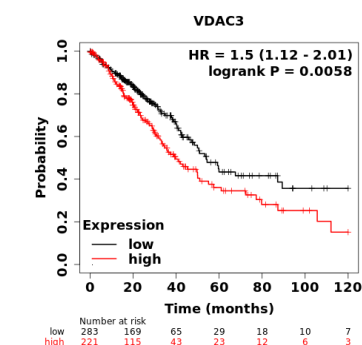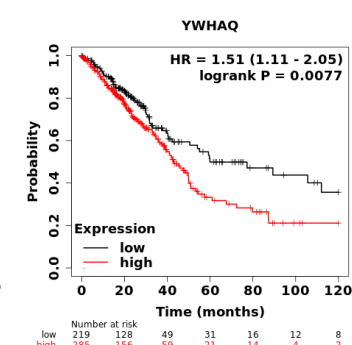

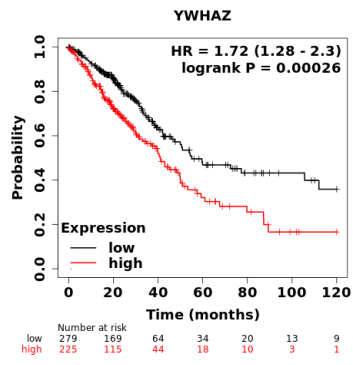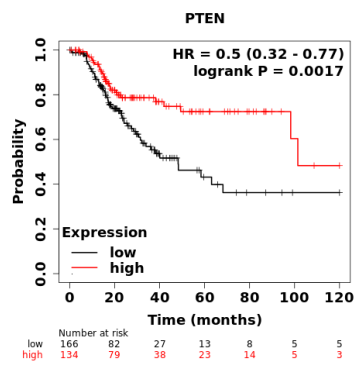

Supplement: Supplementary file 1 [file DataSheet2.PDF]

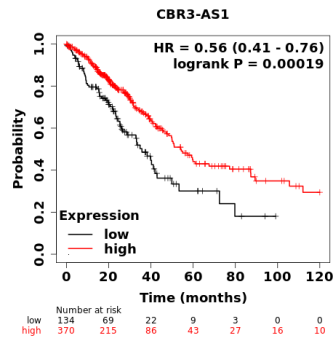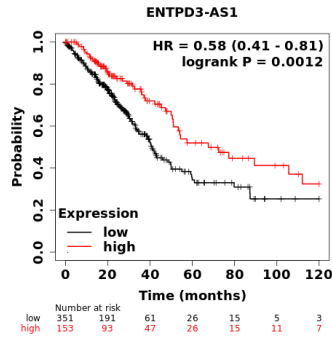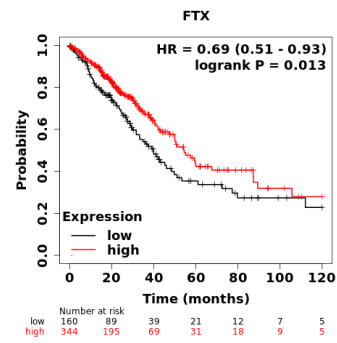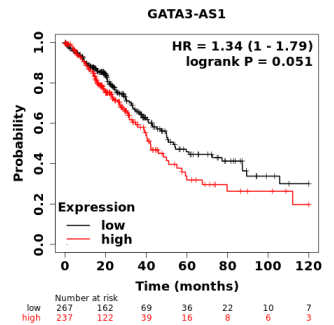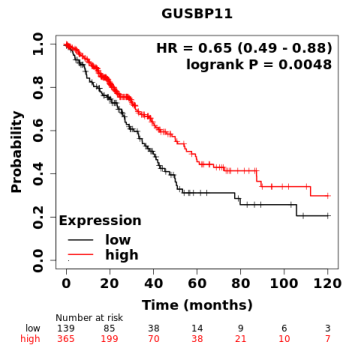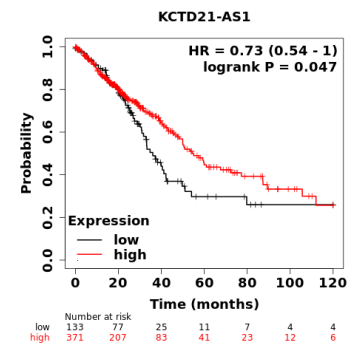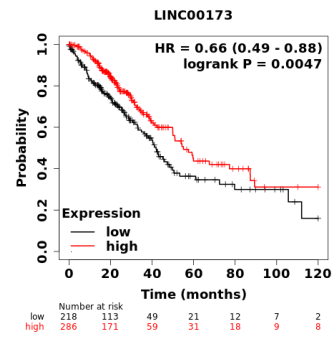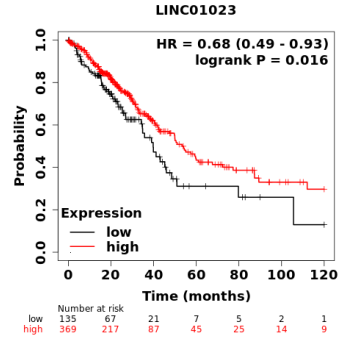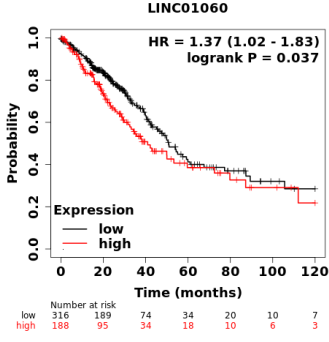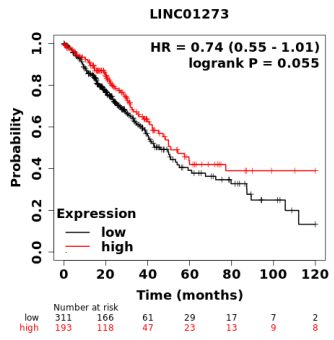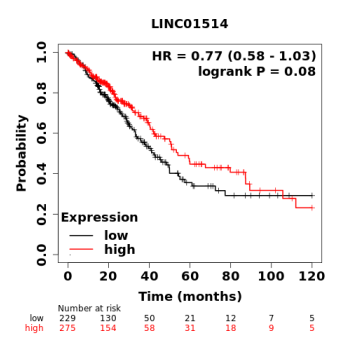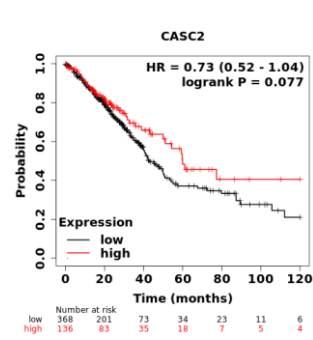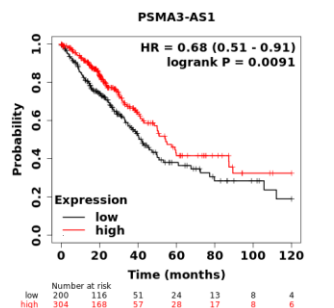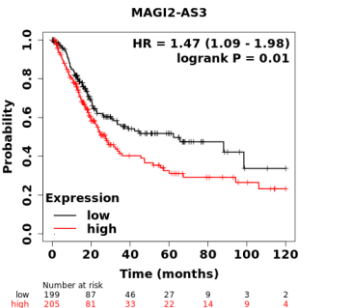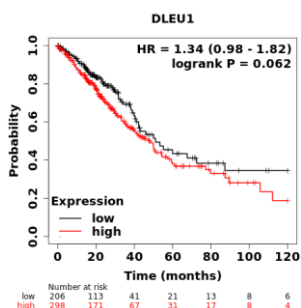

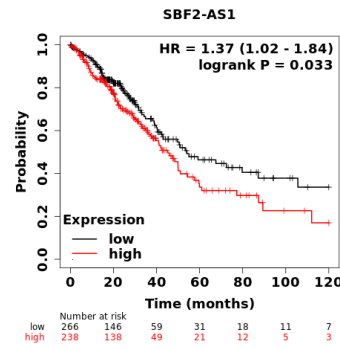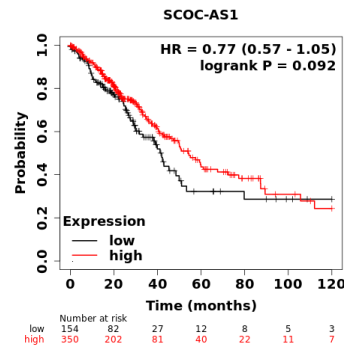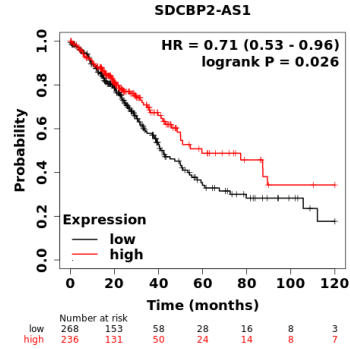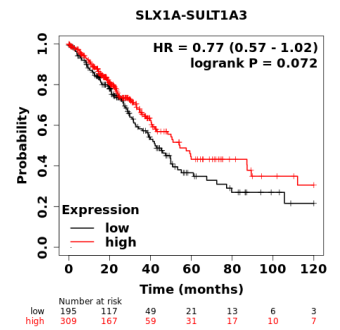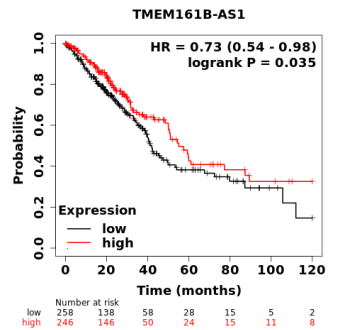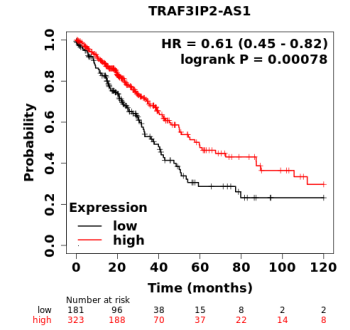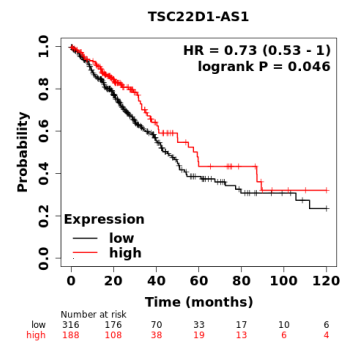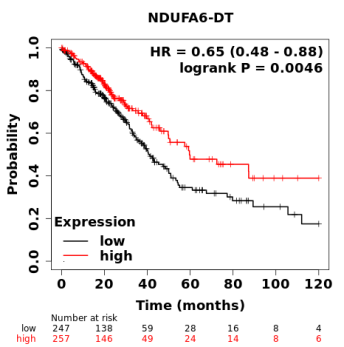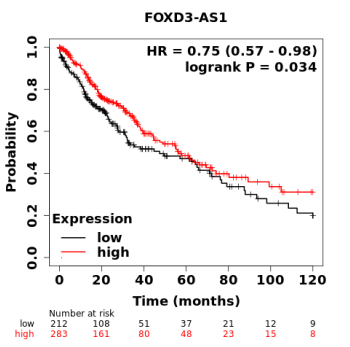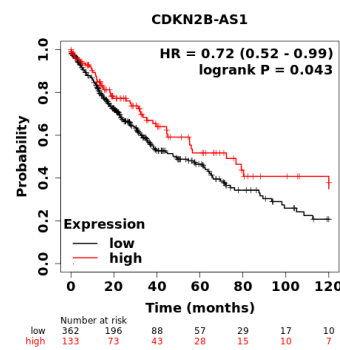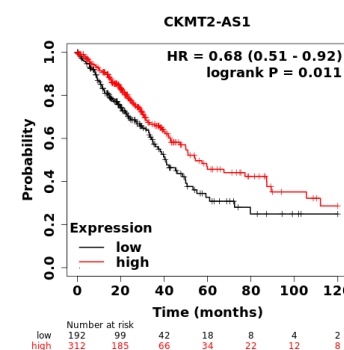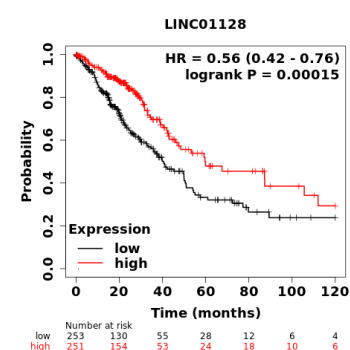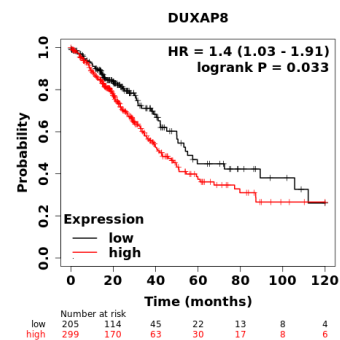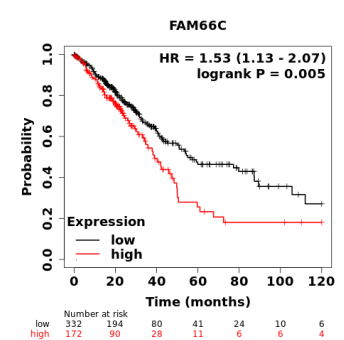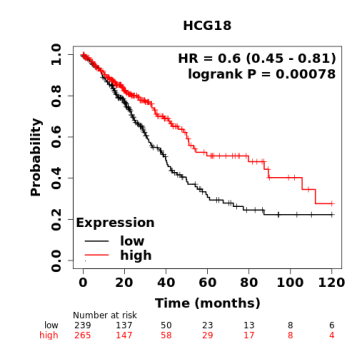

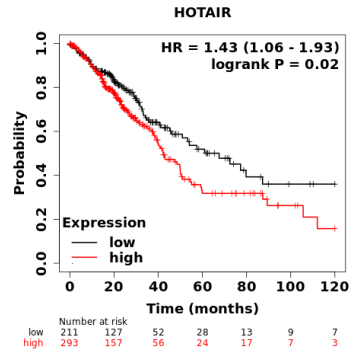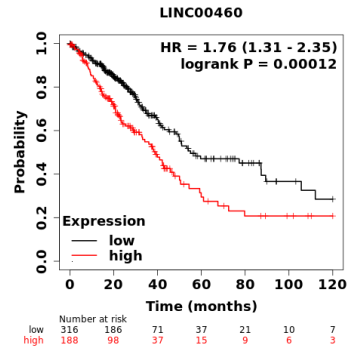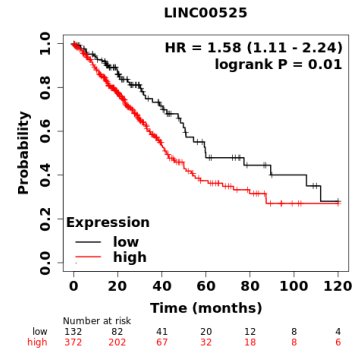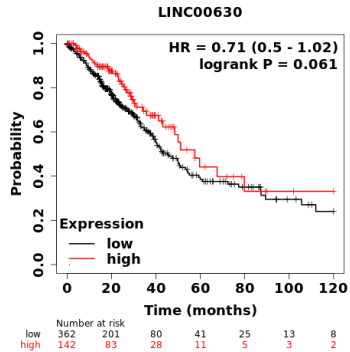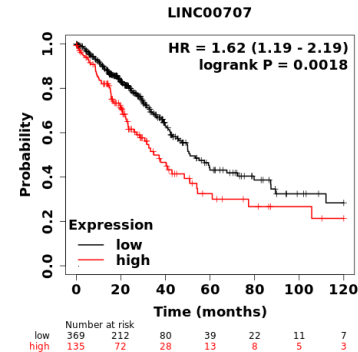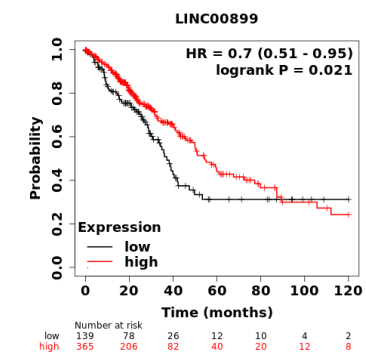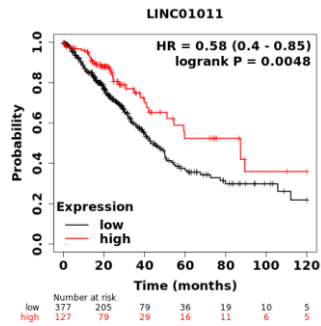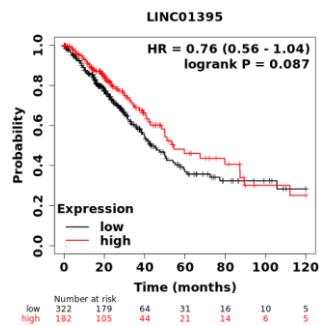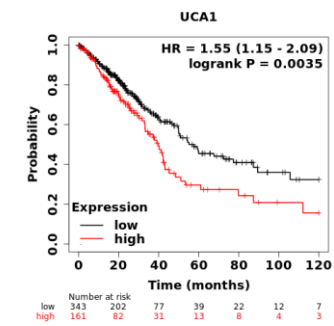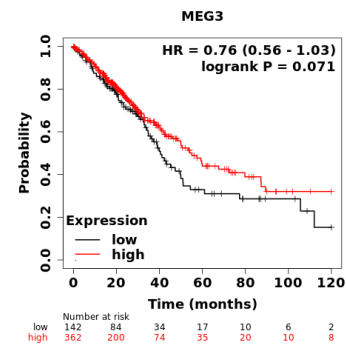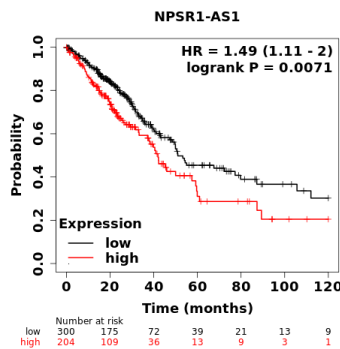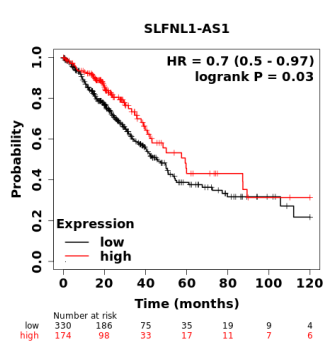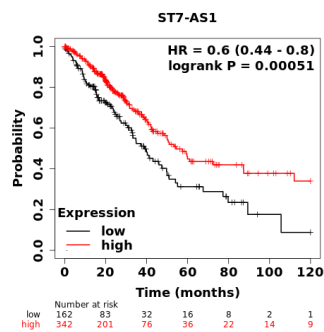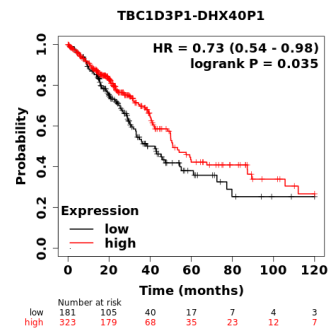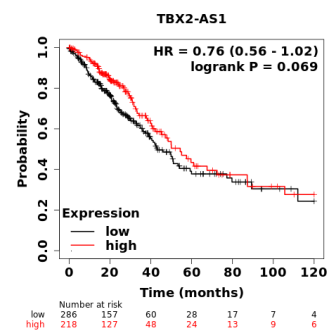

Supplement: Supplementary file 3 [file DataSheet1.PDF]
