# Supplementary material for: Analysis of the lncRNA–miRNA–mRNA Network Reveals a Potential Regulatory Mechanism of EGFR-TKI Resistance in NSCLC
Source: Front Genet. 2022 Apr 29;13:851391. doi: 10.3389/fgene.2022.851391 (PMC9099042; doi:10.3389/fgene.2022.851391)
Supplement: Supplementary file 2 [file Table1.pdf]

Table S1. Several EGFR-TKI resistant datasets

Dateset	GSE71587		GSE33658		GSE23206	
Type	EGFR-TKI sensitive	drug resistance	EGFR-TKI sensitive	drug resistance	EGFR-TKI sensitive	drug resistance
LINC01128	146.3681	136.3808	3.760905	3.44292	223.5167	138.8667
PTEN	2065.664	2011.8965	7.021424	6.873691	4862.157	4525.836
